# High Molecular Weight Chitosan from Shrimp Shells: Synthesis of *Para*-Substituted Schiff Bases with Selective Leishmanicidal Activity and Application in CO_2_/O_2_-Selective Films

**DOI:** 10.3390/polym18111397

**Published:** 2026-06-04

**Authors:** Andrés Alejandro Yánez-Crespo, Christian David Alcívar-León, Pablo Mauricio Bonilla-Valladares, Trosky Germán Yánez-Darquea, Jorge Heredia-Moya, Luciana Juncal, Fabiana Cabrera, María José Andrade-Cuvi, Carlota Moreno-Guerrero, Sonia E. Ulic

**Affiliations:** 1 Facultad de Ciencias Químicas, Universidad Central del Ecuador, Francisco Viteri y Gilberto Sobral s/n, Ciudad Universitaria, Quito 170129, Ecuador; aayanezc@uce.edu.ec (A.A.Y.-C.); pmbonilla@uce.edu.ec (P.M.B.-V.); tgyanez@uce.edu.ec (T.G.Y.-D.); 2 Centro de Investigación Biomédica (CENBIO), Facultad de Ciencias de la Salud Eugenio Espejo, Universidad UTE, Quito 170527, Ecuador; 3 Instituto de Física La Plata (IFLP), UNLP-CONICET, Departamento de Física, Facultad de Ciencias Exactas, Universidad Nacional de La Plata, Diagonal 113 e/63 y 64, La Plata 1900, Argentina; ljuncal@fisica.unlp.edu.ar (L.J.); cabrera@fisica.unlp.edu.ar (F.C.); 4 Laboratorio de Investigación en Ingeniería en Alimentos (LabInAli), Departamento de Ingeniería en Alimentos, Colegio de Ciencias e Ingenierías, Universidad San Francisco de Quito USFQ, Quito 170901, Ecuador; mjandrade@usfq.edu.ec; 5 Centro de Investigación de Alimentos (CIAL), Facultad de Ciencias de la Ingeniería e Industrias, Universidad UTE, Quito 171029, Ecuador; cmoreno@ute.edu.ec; 6 CEQUINOR (UNLP-CONICET), Facultad de Ciencias Exactas, Universidad Nacional de La Plata, Bv. 120 N° 1465, La Plata 1900, Argentina; sonia@quimica.unlp.edu.ar

**Keywords:** chitosan, natural polymer, condensation reactions, cytotoxicity, film, packaging material

## Abstract

*Penaeus* sp. shells (shrimp) were used to extract chitosan using acid and basic treatments, which were characterized by IR spectroscopy, Raman spectroscopy, potentiometric titration, and elemental analysis. The degrees of deacetylation were determined to be 71.8%, 75.6%, 53.4%, and 68.6%, respectively. Likewise, viscosimetry measurements were carried out, determining an average molecular weight of chitosan **1** of 1521467.919 (g/mol). The obtained chitosan was used as a substrate in condensation reactions with 10 *para*-substituted benzaldehydes. The products obtained were characterized by IR, Raman, and ^1^H-NMR spectroscopy, AE (Elemental Analysis), TGA (Thermogravimetric Analysis), and DSC (Differential Scanning Calorimetry). For the obtained polymers, biological assays of cytotoxicity using RAW macrophage cells and leishmanicidal activity on promastigotes of *Leishmania mexicana* were performed. The results show that the synthesized products do not present in vitro cytotoxicity, and that **1** (Chitosan) and **3i** (Schiff Base) present leishmanicidal activity. Selected derivatives were incorporated into polyvinyl alcohol-based films and evaluated for surface topography and gas permeability. AFM revealed nanometric roughness patterns, while gas exchange studies demonstrated selective CO_2_/O_2_ permeability, supporting passive modified atmosphere formation in packaged carrots. Mechanical characterization revealed that the incorporation of Schiff base derivatives significantly influences tensile strength and flexibility, with certain films exhibiting enhanced elongation and mechanical performance compared to pure PVA, highlighting their potential for packaging applications. These findings confirm that chemical functionalization enhances the versatility of chitosan, allowing the design of tailored biopolymers. The synthesized derivatives show promising characteristics for the development of biodegradable films with potential applications in food packaging and antiparasitic material development.

## 1. Introduction

The seafood processing industry generates large volumes of waste, primarily shrimp exoskeletons, which account for 35% to 45% of the total weight of dried shrimp, with approximately 30% to 40% consisting of chitin. Due to its *beta*-glycosidic linkages, this polysaccharide is indigestible by humans and is consequently discarded as waste. Its utilization has been the focus of various studies, including the production of chitosan through chemical treatments [1,2].

Chitosan is a biodegradable, biocompatible polymer with antimicrobial properties, making it attractive for applications in medicine, agriculture, food, and the pharmaceutical industry. Its ability to undergo chemical modifications enables the development of new materials, including biodegradable packaging that could replace widespread plastic usage. In this context, the chemical functionalization of chitosan via reactions with aldehydes to form Schiff bases offers a promising approach for obtaining polymers with enhanced properties [3]. Proper functionalization and characterization allow the development of biodegradable films with potential applications in food packaging. These films have been studied for their effectiveness in food preservation [4]. Various techniques, such as casting, coating, spraying, and electrospinning, have been explored for developing biodegradable chitosan-based films. The production of chitosan fibers with diameters below 1 μm from natural sources expands their potential applications in the sustainable packaging industry. The extraction and chemical modification of chitosan from shrimp waste present a promising strategy for developing novel biopolymers applicable in manufacturing biodegradable materials [5].

In this sense, chitosan was functionalized with ten different benzaldehydes. The resulting compounds were characterized, and various properties, including thermal characteristics, were evaluated. Functionalization was confirmed through IR/Raman spectroscopy, while elemental analysis provided insights into the chemical composition of the modified chitosan derivatives.

Leishmanicidal activity and cytotoxicity were evaluated to explore the potential of the synthesized derivatives within a specific antiparasitic context. Leishmaniasis is a neglected tropical disease with significant global impact, and current treatments are often limited by toxicity and drug resistance [6]. Chitosan and its derivatives have attracted attention due to their biocompatibility and reported antileishmanial activity, as well as their potential to improve therapeutic performance [7]. Therefore, in this study, leishmanicidal activity was investigated as a targeted biological property rather than as a general biomedical claim. The development of films from the functionalized derivatives enabled the assessment of surface roughness and gas kinetics (CO_2_ and O_2_) for potential use in packaging materials. Gas confinement and permeability studies revealed that the composition of the synthesized product plays a significant role in its functional properties. Recent studies have demonstrated the potential of chitosan–Schiff base derivatives for the development of active and biodegradable packaging materials due to their tunable physicochemical, thermal, and biological properties. Several authors have reported improvements in antimicrobial activity, mechanical performance, and gas barrier properties through chemical functionalization of chitosan with aromatic aldehydes and related compounds. However, most previous studies have focused either on biological activity or on packaging applications separately [8,9,10,11,12,13].

In contrast, the present work combines the synthesis of para-substituted chitosan–Schiff base derivatives, evaluation of selective leishmanicidal activity, and application of the modified polymers in CO_2_/O_2_-selective films, integrating structural, thermal, surface, mechanical, and permeability analyses. To the best of our knowledge, this combined approach has not been previously reported for chitosan-based Schiff base systems derived from shrimp shell waste.

## 2. Materials and Methods

### 2.1. Materials

Shrimp shells (*Penaeus* sp.) were obtained from a local seafood processing source. Hydrochloric acid (HCl), sodium hydroxide (NaOH), acetic acid, ethanol, and all benzaldehydes (**2a–j**) were purchased from commercial suppliers and used without further purification. Polyvinyl alcohol (PVA) was used as received. Commercial chitosan (Qc) was used as a reference material for comparison purposes (BOC Sciences, Shirley, NY, USA; Catalog No. B1370-187887; CAS No. 9012-76-4). For biological assays, Dulbecco’s Modified Eagle Medium (DMEM), fetal bovine serum (FBS), penicillin–streptomycin, MTT reagent, dimethyl sulfoxide (DMSO), and Amphotericin B were obtained from standard suppliers. RAW 264.7 macrophage cells and Leishmania mexicana promastigotes were used as biological models.

### 2.2. Processing of Chitin from Shrimp Shells to Obtain Chitosan

#### 2.2.1. Grinding and Screening

The shrimp’s exoskeletons were cleaned of any remaining biological content. Afterwards, the heads and legs were removed, leaving only the shells to dry at room temperature for 3 days. The dried shells were ground up with a blade mill to produce a powder, which was then passed through a 250-micron sieve and used as a raw material to obtain chitosan.

#### 2.2.2. Deproteination and Demineralization

For the demineralization process, 46.10 g of sieved powder was placed in a 500 mL beaker with 230.50 mL of a 1:5 (*w*/*v*) solution of 2 N HCl, and the suspension was stirred for 2 h at room temperature. The solid was filtered and washed with abundant water until the pH was neutral. The product was then dried in an oven at 50–60 °C for 24 h [14]. Subsequently, 33.13 g of the demineralized product was weighed, and 331.30 mL of a 1:10 (*w*/*v*) 2 M NaOH solution was added while stirring the suspension at 100 °C for 3 h. The product obtained was filtered and washed with abundant water until the pH was neutral. Finally, the product was dried at 50–60 °C for 24 h to afford 19.10 g of chitin [14].

#### 2.2.3. Deacetylation

In a round-bottom flask, a mixture containing 19.10 g of the demineralized and deproteinized powder and a 1:10 *w*/*v* solution of 50% NaOH was refluxed for 2 h. Then, the solid was filtered and washed with abundant water until the pH reached neutrality. Finally, the product was dried in an oven at 50–60 °C for 24 h to afford 15.68 g of chitosan **1**.

### 2.3. Characterization of Chitosan from Shrimp Shells

#### 2.3.1. Average Molecular Weight by Capillary Viscosimetry

The viscosity was measured using an Ostwald viscometer with a 0.3 mm capillary of 250 mm length submerged in a temperature-controlled bath (±0.01 °C). Chitosan was dissolved in 1% *w*/*v* acetic acid to prepare a 0.01 g/mL stock solution. Dilutions of 8.0, 6.0, 4.0, and 2.0 mg/mL were made using this stock solution. After charging a solution in the viscometer, it was immersed in the thermal bath, and the time it took to flow through the capillary tube was recorded. This procedure was performed for each solution, including the solvent. Additionally, each solution’s relative density was determined using the pycnometer method [15].

The molecular weight of chitosan was determined by capillary viscometry, using the intrinsic viscosity [ɳ]. The experimental data obtained were used to calculate the dynamic, specific, and relative viscosity using consecutively the equations detailed in the supplementary material (Equations (S1)–(S4)). Using the reduced viscosity calculated with S4, a linear regression (Figure S7) was performed to obtain the intrinsic viscosity, which is related to the molecular weight of the polymer according to the Mark–Houwink equation.

(1) $$\left[ɳ\right]=KM_{v}^{a}$$

K and *a* are empirical constants that depend on the nature of the polymer and the solvent. In this case, K is equal to 0.00474, and *a* is equal to 0.72 [15].

#### 2.3.2. Degree of Deacetylation (DD) by IR and Raman Spectroscopy

The chitosan IR spectrum was measured from 400 cm^−1^ to 4000 cm^−1^ using a VARIAN 660-IR/FT-IR Spectrometer with 1.92 cm^−1^ resolution and 64 scans, using the KBr disk technique (2.0 mg of compounds and 10 mg of KBr). To determine the degree of deacetylation (DD), the area under the curve of the absorption bands at 1320 cm^−1^ and 1420 cm^−1^ was measured, and DD was calculated using Equations (2) and (3) [16].

(2) $$\frac{A_{1320}}{A_{1420}}=0.3822+0.03133\;DA$$

(3) DD=100−DA 

A_1320_/A_1420_ represents the adsorption areas ratio, DA is the chitosan acetylation percentage, and DD is the deacetylation percentage [16,17].

The Raman scattering spectrum of chitosan was recorded in the spectral range of 100–3500 cm^−1^ in standard Pyrex capillaries (2.5 mm, i.d), using a Perkin–Elmer FT-Raman RFs 100/f spectrometer with Nd/YAG laser excitation radiation (spectral resolution of 4 cm^−1^) of the 1064 nm line. The spectrum obtained was analyzed using SpectraGryph version 1.2. The biopolymer’s deacetylation degree was determined by comparing signal heights at 1591 cm^−1^ and 896 cm^−1^, using Equation (4) [18].

(4) $$\frac{H_{1591}}{H_{896}}=0.014\;DD-0.86$$

H_1591_ and H_896_ represent the height of the signals in the Raman spectra at 1591 cm^−1^ and 896 cm^−1^, respectively. DD is the percentage of deacetylation [19].

#### 2.3.3. Degree Deacetylation (DD) by Potentiometric Titration Method

The degree of deacetylation of chitosan was measured with a METLER TOLEDO model LE438 potentiometer. The assay was performed in duplicate, and 0.2533 g and 0.2514 g of chitosan were weighed for each assay. The sample was dissolved in 20.0 mL of 0.3270 M HCl that had been previously titrated. The mixture was then titrated with the same NaOH solution (0.1046 M), and the pH was measured after each addition of 2.0 mL of the hydroxide solution while stirring continuously. Subsequently, a titration curve of pH vs. mL of added NaOH was constructed. Equation (5) was used to determine the degree of deacetylation of chitosan, which is directly related to the concentration of free amino groups in the molecule [15].

(5) $$\%{NH}_{2}=\frac{16.1*(Y-X)}{w}*f$$

Y is the main inflection point, and X is the minor inflection point, expressed in mL, *f* is the molarity of the NaOH solution, w is the mass in grams of the sample, and 16.1 is a factor associated with the type of study protein.

#### 2.3.4. Degree of Deacetylation (DD) by Elemental Analysis (EA)

The elemental analysis of chitosan was performed using a Vario MICRO Cube Brand elemental analyzer (Elementar Analysensysteme GmbH, Langenselbold, Germany). The percentage of deacetylation of chitosan was determined using Equation (6) [20], where W_C/N_ represents the ratio of the percentage of carbon and nitrogen in the chitosan sample.

(6) $$DD=100*\left(4-0.583093*W_{\frac{C}{N}}\right)$$

### 2.4. Synthesis and Characterization of Schiff Base of Chitosan

#### 2.4.1. General Procedure for the Synthesis of Chitosan Schiff Base Derivatives

**Scheme 1:**
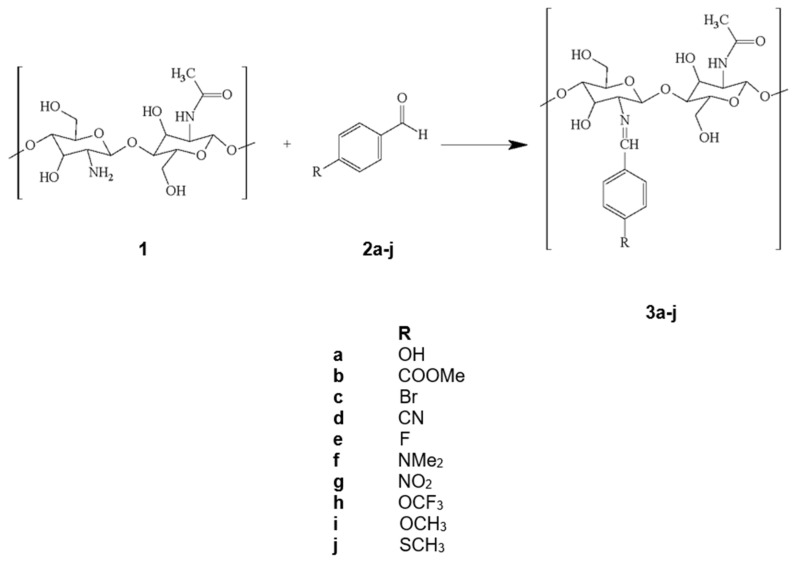
Synthesis of benzaldehyde-functionalized chitosan: (**1**) chitosan molecule; (**2a–j**) aldehyde; (**3a–j**) Schiff Base.

The Schiff bases were synthesized using the procedure previously reported [3,21], as shown in Scheme 1. The benzaldehyde **2a–j** stoichiometry ratio to the free amino group used for the synthesis was 1.5:1 (in mol) in all reactions (Table 1). An example of the stoichiometric calculation is detailed in the Supplementary Materials.

Chitosan was added to a 100 mL round-bottom flask, followed by 25 mL of 0.15 M acetic acid. The mixture was stirred for 24 h until the chitosan was dissolved entirely. Then, the required amount of benzaldehyde (**2a–j**) was dissolved in 10 mL of ethanol, added to the chitosan solution, and the mixture was refluxed with constant stirring for 24 h. The Schiff base obtained (**3a–j**) was then filtered, washed with ethanol to eliminate unreacted benzaldehyde, and dried for 24 h at 50–60 °C.

#### 2.4.2. Characterization by Vibrational Spectroscopy: IR and Raman

The FTIR spectra were acquired using the KBr disk technique (2.0 mg of compounds and 10 mg of KBr) on a VARIAN 660-IR/FT-IR Spectrometer with a resolution of 2 cm^−1^ and 64 scans from 400 cm^−1^ to 4000 cm^−1^. The Raman spectra of all compounds were measured using a Perkin–Elmer FT-Raman RFs 100/f spectrometer with standard Pyrex capillaries (2.5 mm, i.d) and a line excimer light source of 1064 nm from an Nd/YAG laser (spectral resolution of 4 cm^−1^) in a spectral region of 100–3500 cm^−1^. All spectra were analyzed using SpectraGryph version 1.2.

#### 2.4.3. ^1^H NMR Spectroscopy

^1^H NMR spectra of chitosan and selected Schiff base derivatives were acquired on an Oxford Instruments Pulsar benchtop NMR 60 MHz Spectrometer. Samples were prepared by dissolving the polymers in 1% HCl/D_2_O (*v*/*v*) at 60 °C and then analyzed at the instrument temperature (~37 °C) [22]. Spectra were collected using standard pulse sequences with 128 scans. Due to the polymeric nature of the samples and the experimental conditions employed, the spectra were used for qualitative structural analysis rather than quantitative determination of substitution degree.

#### 2.4.4. Characterization by Differential Scanning Calorimetry (DSC)

Differential scanning calorimetry analysis was carried out in a Shimadzu DSC-50 instrument, using aluminum crucibles, in the temperature range of 20 °C to 500 °C, with a heating rate of 10 °C/min and nitrogen flow of 20 mL/min.

#### 2.4.5. Characterization by Thermogravimetric Analysis (TGA)

A Shimadzu model TGA-50H thermobalance and platinum crucibles were used. The heating rate was 10 °C/min, and the nitrogen flow rate was 20 mL/min, in a temperature range of 20 °C to 700 °C.

#### 2.4.6. Characterization by Elemental Analysis (EA)

A Vario MICRO cube elemental analyzer was used for EA. Helium was employed at a pressure of 1.200–1.500 bar with 99.995% purity and oxygen at 2 bar with 99.995% purity. Cold leak tests were carried out, and the equipment was programmed for combustion at 1150 °C and reduction at 850 °C. A 2.0 mg sample was used for all determinations, and a sulfanilamide standard was used as a control.

### 2.5. Cell Viability of Schiff Bases of Chitosan

Compounds **1** and **3a–j** were tested for in vitro viability in the RAW 264.7 cell line using the MTT colorimetric assay [23]. RAW 264.7 cells were maintained in Dulbecco’s modified Eagle medium supplemented with 10% fetal bovine serum and 100 IU/mL penicillin + 100 ug/mL streptomycin at 37 °C in a 5% CO_2_ atmosphere. The medium was renewed once a week.

To test cell viability, 50.000 cells per well were placed on a 96-well plate with a final volume of 100 µL. After 2 h of incubation to fix the cells to the plate’s bottom, the compounds were added at a final 100 µg/mL concentration. Following 24 h of incubation, 10 µL of MTT (5 mg/mL in PBS) was added to the plate and incubated for 2 h. After centrifugation at 4400 rpm for 10 min at room temperature, the media was removed, and 100 µL of DMSO was added to dissolve the formazan. In a plate-reading spectrophotometer, the absorbance was measured at 570 nm, and a reference wavelength of 630 nm was used to subtract the background. Data were analyzed using the statistical software GraphPad Prism 7.02 (GraphPad Software, Corp., San Diego, CA, USA).

### 2.6. Evaluation of Leishmanicidal Activity of Schiff Bases of Chitosan

Leishmanicidal activity in *Leishmania mexicana* promastigotes was determined using a colorimetric MTT assay [23]. The parasites were cultivated at 25 °C in Schneider’s Drosophila medium supplemented with 10% fetal bovine serum. The culture media were renewed every four days, and the parasite density was determined using a Neubauer chamber.

In order to evaluate parasite viability, 1 × 10^6^ parasites per well were added to each well of a 96-well plate, followed by the addition of compounds **1** and **3a–j** at a final concentration of 50 µg/mL with a total volume of 200 µL. Positive and negative controls were 1 µM of Amphotericin B (Gibco), untreated parasites, and 2.0% acetic acid. After 24 h of incubation at 25 °C, 20 µL of MTT solution (5 mg/mL in PBS) was added to each well. The plate was incubated in darkness for an additional 2 h at the same temperature; then it was centrifuged at 4400 rpm for 10 min, and the culture media were removed. Finally, 50 µL of DMSO was added to each well, followed by shaking for 5 min, and the absorbance was measured at 570 nm in a Cytation 5 (BioTek) microplate reader.

Background subtraction was carried out using a reference wavelength of 630 nm. Data analysis was performed using GraphPad Prism 7.02, a statistical program.

### 2.7. Development and Characterization of Films Based on Schiff Bases ***3a–j*** with PVA

#### 2.7.1. Preparation Process of Blends of Chitosan Films and **3a–j**–PVA

Solutions (0.75% *w*/*v*) of chitosan **1**, commercial chitosan Qc, and Schiff bases **3a–j** were prepared using 60% acetic acid as the solvent. The mixture was stirred for 24 h at room temperature until completely dissolved. For each film, 4 g of each solution was mixed with the same amount of an aqueous solution containing 8% *w*/*v* PVA. The mixture was stirred for 5 min, poured onto a flat surface, and left to dry at room temperature. Although dilute acetic acid solutions (1–5%) are commonly used for chitosan dissolution, a higher concentration was employed in this study to ensure complete solubilization of the high-molecular-weight chitosan obtained. Possible partial hydrolysis effects cannot be completely excluded and should be considered as a limitation of the present study.

#### 2.7.2. Atomic Force Microscopy of Films

The roughness and topography of the films were determined using a Park system model NX10 atomic force microscope (AFM). The equipment was programmed to operate in contact mode, with a tip-to-sample distance ranging from 100 to 180 nm. A micro lever with a silicon nitride tip was used for the measurements. Samples measuring 1 cm × 1 cm were cut from each film for surface mapping. AFM scans were acquired over areas of 2.5 µm × 2.5 µm. Due to sample and measurement limitations, one representative scan was analyzed for each film; therefore, the roughness values should be interpreted as comparative surface indicators rather than statistically exhaustive roughness measurements. Each mapped surface’s topographic roughness parameter (Ra) was considered to infer the interaction dynamics of the CO_2_/O_2_ confined atmosphere due to carrot transpiration with the film surface.

### 2.8. Evaluation of CO_2_/O_2_ Concentration Using Films Based on Schiff Base with PVA as Simulation Packing Material

Minimally processed carrots were placed in 250 mL glass jars covered with films selected for their leishmanicidal properties. Approximately 200 g of baby carrots, obtained from a local market, were placed in each jar and sealed with the corresponding film. This assay was performed on the films for compounds **1**, **3a**, and **3b**. The headspace gas composition of the packaged samples was analyzed using a digital gas analyzer (Mocon PBI Dansensor, CheckPoint CO_2_/O_2_). The jars were stored at 7 °C, and gas concentrations (% CO_2_/O_2_) were monitored for 240 min. All experiments were performed in triplicate.

### 2.9. Mechanical Properties of Films

The mechanical properties of the films were evaluated using a universal testing machine (Shimadzu). Rectangular specimens were prepared from the films and tested at room temperature under a constant crosshead speed of 5 mm/min. The maximum tensile strength (N/mm^2^), elongation at break (%), and displacement (mm) were recorded. At least two replicates were analyzed for each sample, and the results are reported as mean ± standard deviation. Due to limitations in material availability, some samples were analyzed in duplicate. Therefore, the mechanical data should be interpreted as comparative rather than definitive quantitative measurements.

## 3. Results and Discussion

### 3.1. Chitosan Obtaining

The chitosan was extracted from treated shrimp waste. The first step was a demineralization process, in which the pulverized shrimp shells were treated with hydrochloric acid at room temperature. During this step, the inorganic matter, mainly CaCO_3_, was eliminated, and 71.87% of the initial weight was recovered. The demineralized chitin was then deproteinized in a hot 50% sodium hydroxide (NaOH) solution. This procedure removed the proteins found in the shrimp shells, allowing 41.43% of the chitin to be recovered. The last step in producing chitosan is deacetylation. This step involves a basic hydrolysis of the acetamide groups positioned in the C2 position of the chitin, allowing the amine group to be released. We recovered 15.68 g of chitosan **1** from 46.10 g of shrimp shells, representing 34.01%, comparable with previously published values [14,24].

### 3.2. Molecular Weight by Capillary Viscosimetry of Chitosan

One method for calculating the molecular weight of chitosan involves measuring the viscosity of different concentrations of this biopolymer [25]. To estimate the average molecular weight of chitosan **1**, the intrinsic viscosity [η] was determined for different chitosan solutions. As expected, as the solution concentration increases, the time it takes to flow through the capillary of the Ostwald viscometer (Table 2) also increases. Equation (S1) (Supplementary Materials) was used to calculate the dynamic viscosity of each diluted chitosan solution based on the average times and density. The viscometer’s constants (k1 and k2) were 0.018530899 (cm^2^/s^2^) and 0.033499762 (cm^2^), respectively [15,26]. The calculated dynamic viscosities were then plotted against the concentrations of chitosan solutions, allowing for the determination of intrinsic viscosity through linear regression analysis, which provides insights into the polymer’s molecular weight characteristics [27]. The calculated intrinsic viscosity was 133.97 (g/cm^3^), and the average molecular weight was estimated by applying the logarithmic Mark–Houwink equation (Equation (1)) to be 1,521,467.919 (g/mol) or 1521.5 kDa, which indicates that the chitosan obtained is of high molecular weight [28].

The average molecular weight is directly related to the purity of chitosan and its potential applications [29]. However, the relationship between molecular weight and biological activity is not straightforward and depends on the specific application. For example, higher molecular weight chitosan is often associated with improved film-forming ability and mechanical properties, while lower molecular weight fractions may enhance solubility and biological interactions. The high average molecular weight obtained for chitosan 1 (1521.5 kDa) is comparable to the reported value for commercial chitosan Qc (874 kDa) [28,30,31]. The literature indicates that the molecular weight of chitosan depends on the extraction and deacetylation conditions, typically ranging from 10^5^ to 10^6^ g/mol [32].

### 3.3. Degree of Deacetylation

The degree of deacetylation of chitosan is a critical parameter that influences its solubility and biocompatibility and has a considerable impact on its physicochemical and mechanical properties and biological activity, making it an essential factor in various applications such as drug delivery, wound healing, and food preservation [33]. The degree of deacetylation of chitosan refers to the proportion of β-(1-4) *D*-glucosamine units (deacetylated units) present in the biopolymer chain after acetyl groups are removed from chitin during its conversion to chitosan [34]. Higher degrees of deacetylation generally enhance the solubility of chitosan in aqueous solutions, facilitating its use in various biomedical and pharmaceutical applications where improved interaction with biological systems is desired. Various methods can be used to determine the degree of deacetylation in chitosan [35], including titration [36], infrared spectroscopy [37], Raman spectroscopy [18], and elemental analysis [20], which were employed in this study to evaluate the degree of deacetylation of **1**. Each technique provides unique insights into the biopolymer’s structure and properties while ensuring accurate assessment for practical applications [33].

#### 3.3.1. Determination of the Percentage of Deacetylation by IR and Raman Vibrational Spectroscopy

Infrared (IR) spectroscopy has been widely used to characterize the chemical composition of chitosan with diverse degrees of deacetylation [16,29]. This technique identifies functional groups and provides insights into the structural changes that occur as chitosan is processed. Figure S1 (Supplementary Materials) shows the experimental FT-IR and Raman spectra of **1**, and Table S1 (Supplementary Materials) describes the absorption bands associated with the main functional groups present in the biopolymer.

The absorption bands observed in the IR spectrum at 3430 cm^−1^ and 3369 cm^−1^ suggest the existence of hydroxyl groups and are attributed to the stretching vibrational modes. Similarly, the shoulder-shaped IR absorption bands at 3279 cm^−1^ and 3104 cm^−1^ have been assigned to the asymmetric and symmetric stretching of the N-H bond. The band at 2919 cm^−1^ (2933 cm^−1^ in Raman) was attributed to the stretching of the -CH_3_ group. Likewise, the relatively weak absorption bands at 2878 cm^−1^ and 2854 cm^−1^ (2884 cm^−1^ and 2867 cm^−1^ in Raman) are assigned to the asymmetric and symmetric stretching of the -CH_2_ group.

The amide’s carbonyl group (C=O) shows a strong IR absorption band at 1653 cm^−1^ (1654 cm^−1^ in Raman). The absorption band observed at 1420 cm^−1^ was assigned to the deformation of the -CH_3_ group. The bands within the spectral region of 1379 cm^−1^ to 1154 cm^−1^ are attributed to the combined frequencies of several vibrational modes, including δ (CH), ν (C-N), δ (OH…O), γ (CH_2_), and δ (CH_3_). Likewise, the absorption band at 1074 cm^−1^ (1075 cm^−1^ in Raman) is attributed to the asymmetric stretching of the C-O bond in the remaining ether functional group [17,19].

As reported in the literature [16], the spectral bands at 1320 cm^−1^ and 1420 cm^−1^ were selected to determine the degree of deacetylation. The absorbance A_1320_/A_1420_ ratio shows a reduced experimental error across various techniques. This ratio is exclusively influenced by the chemical composition of chitin (or chitosan), regardless of the methodology used, the physical state, or the secondary structural configuration. The result shows that **1** has a deacetylation degree of 71.8%.

On the other hand, for the calculation of the deacetylation degree by Raman spectroscopy, the comparative height measurements of the bands assigned to the vibrations of the amine group and the glycosidic ring at 1591 cm^−1^ and 896 cm^−1^, respectively, were used [18]. These bands offer unique advantages for evaluating the degree of deacetylation in chitosan, including increased sensitivity, rapid analysis, and the potential for quantitative measurements while ensuring sample integrity. The degree of deacetylation of **1** obtained by this method was 75.6%, similar to that obtained using IR.

#### 3.3.2. Determination of Deacetylation Percentage by Potentiometric Titration

Figure S2 (Supplementary Materials) shows the potentiometric titration results for chitosan. The titration curve shows two inflection points, evidenced by the first derivative criterion.

The amount of acid required to protonate the amino groups of chitosan corresponds to the difference between the two inflection points in the titration curve; the concentration of these is determined using Equation (5) [15]. This results in a deacetylation percentage (%NH_2_) of 53.40 ± 0.28%.

#### 3.3.3. Determination of the Deacetylation Percentage by Elemental Analysis

The results of the elemental analysis of chitosan are shown in Table S2 (Supplementary Material). Equation (5) considers the C/N composition ratio of chitosan to establish the degree of deacetylation (DD), which was 68.62%.

Table 3 presents the deacetylation percentages (DD) calculated using IR and Raman vibrational methods, potentiometric titration, and elemental analysis of chitosan. The results of DD by potentiometric titration and elemental analysis show a divergence from those obtained by IR and Raman spectroscopy. The inherent difficulty in solubilizing the biopolymer could affect the potentiometric titration assay, which presents the lowest DD value [19]. Likewise, although the elemental analysis shows percentages of C and N in chitosan extracted from shrimp shells similar to those of commercial chitosan Qc, the elemental analysis technique has a limited linear range at low concentration levels, affecting the DD value [20].

On the other hand, the method to determine DD by Raman spectroscopy, as it does not require preparation or dissolution of the chitosan samples and has a simple calculation procedure that does not depend on subjectivity, is suggested as the most convenient method to determine the degree of deacetylation. Furthermore, the value obtained is comparable with the deacetylation range of commercial chitosan [19].

### 3.4. DSC/TGA Thermogravimetric Analysis for Chitosan

Chitosan 1 was characterized using Differential Scanning Calorimetry (DSC) and Thermogravimetric Analysis (TGA), which are complementary techniques. These methods provide insights into chitosan’s thermal stability, decomposition behavior, and phase transitions, allowing for a comprehensive understanding of its material properties [38,39,40].

The data acquired from Differential Scanning Calorimetry (DSC) (Figure S3a, Supplementary Materials) show mass loss processes below 100 °C and exhibit an endothermic peak associated with the elimination of reticular water, which corresponds to water molecules that engage in weak interactions, are physically adsorbed, or are held by hydrogen bonds. An exothermic process of around 300 °C is then observed, which could be related to the thermal degradation of chitosan, either by breaking bonds in its polymeric structure or by the decomposition of residual acetylated groups in the sample [41]. The main events observed above 350 °C primarily relate to its thermal decomposition, which is influenced by factors such as the degree of deacetylation and molecular weight [42]. As the temperature continues to rise, further decomposition occurs, forming various volatile products and char residues.

The thermal degradation profile obtained from TGA (Figure S3b, Supplementary Materials) supports these findings. An initial mass decrease (~10%) occurs below 150 °C, linked to the evaporation of the adsorbed moisture. The sharp decline detected around 300 °C reflects the primary thermal breakdown of chitosan, caused by cleavage of the polymeric chain and the release of volatile byproducts. It is also evident that, when temperatures exceed 400 °C, decomposition proceeds more gradually, suggesting the formation of carbonaceous residues [43].

### 3.5. Functionalization of Chitosan

The condensation reactions to form the Schiff bases **3** in the monomeric core were carried out using chitosan from shrimp shells with different *para*-substituted benzaldehydes. For this, a mixture of benzaldehyde **2a–j** and chitosan **1** was reacted, maintaining a 1.5/1 ratio as previously reported [3,21].

The functionalization of **1** through the formation of Schiff bases with various para-substituted benzaldehydes led to the synthesis of ten distinct derivatives. Spectroscopic analyses confirmed the successful formation of imine linkages, with characteristic C=N stretching bands observed between 1642 and 1659 cm^−1^. These chemical modifications demonstrated that the nature of the substituents in Schiff base synthesis directly influences the degree of substitution and, consequently, the resulting biological properties.

### 3.6. Characterization of Chitosan Derivatives ***3a–j***

#### 3.6.1. IR and Raman Spectroscopy

**Figure 1:**
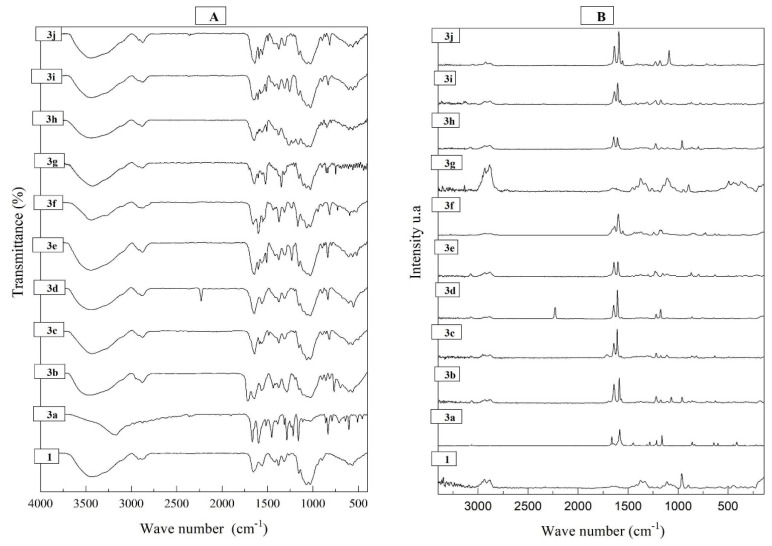
Spectra of chitosan **1** and Schiff bases **3a–j**. (**A**) FT-IR and (**B**) Raman.

The FT-IR and Raman spectra of **1** were compared with those of Schiff bases **3a–j**, showing notable differences in functional groups (Figure 1), which emphasize the structural changes made during the synthesis of these derivatives. The spectral variations confirm the successful formation of Schiff bases.

The bands for **3a–j** in the range 1665–1640 cm^−1^ in both spectra were attributed to the C=N stretching, confirming the formation of the imine group double bond. This Schiff base’s distinctive absorption band is not observed in chitosan **1** as expected [21].

The absorption bands in IR and Raman of the imine group and the benzene ring substituents in Schiff bases **3a–j** are exhibited in Table 4. It is observed that the imine group’s absorption band appears between 1659 and 1642 cm^−1^ in infrared and between 1662 and 1638 cm^−1^ in Raman. However, the substituent on the aromatic ring does not impact the position of these bands. On the other hand, the bands corresponding to the substituents appear in the expected locations, according to the bibliography [21].

In compound **3a**, a weak absorption band at 1218 cm^−1^ and 1213 cm^−1^ in IR and Raman is observed, respectively, and these bands are attributed to the stretching modes of the OH group. Schiff base **3b** shows strong Raman and IR bands attributed to C=O ester stretching at 1716 cm^−1^ and 1719 cm^−1^, respectively. Compound **3c** exhibits an absorption IR band at 1071 cm^−1^, attributed to the stretching of C-Br, which was not observed in Raman. The medium-intensity absorption bands at 2229 cm^−1^ and 2231 cm^−1^ in the IR and Raman spectra, respectively, of **3d** can be attributed to the stretching of the CN group.

The IR spectrum of functionalized chitosan **3e** shows a significant absorption band at 1232 cm^−1^ and a Raman band at 1231 cm^−1^, assigned to C-F bond stretching. In the infrared and Raman spectra, the tertiary amine (Ar-NMe_2_), present in compound **3f,** exhibits weak absorption bands at 1232 and 1247 cm^−1^, which are assigned to the bending C-N-C, respectively. On the other hand, the strong absorption band at 1523 cm^−1^ in the IR spectrum of **3g** is associated with the C-NO_2_ stretching. Meanwhile, the bands observed at 1261 cm^−1^ (IR) and 1223 cm^−1^ (Raman) in **3h** are associated with the stretching of the CF_3_ group. The stretching of the C-O-C group is responsible for the bands at 1255 and 1226 cm^−1^ in the infrared and Raman spectra of functionalized chitosan **3i**, respectively. Finally, the weak absorption bands at 1435 and 1452 cm^−1^ in infrared and Raman spectra, respectively, of **3j** correspond to C-S-C bond stretching [44].

#### 3.6.2. ^1^H-NMR Spectroscopy

The ^1^H NMR spectrum of chitosan (Figure S10) shows signals characteristic of the glucopyranoside backbone between 3.0 and 4.5 ppm. Additionally, a signal near 2.1 ppm is attributed to the methyl protons of residual *N*-acetyl groups. A strong signal at approximately 4.7–4.8 ppm corresponds to residual HOD from the solvent. On the other hand, the spectra of Schiff base derivatives are shown in Figures S11–S18. In this sense, all Schiff base derivatives (**3a**–**3g** and **3i**) displayed new signals in the downfield region between 9.7 and 10.2 ppm, assigned to the azomethine proton (–CH=N–) formed during the condensation reaction with the *para*-substituted benzaldehydes. This signal is absent in chitosan, providing direct evidence of successful functionalization. Additionally, signals in the aromatic region (7.0–8.6 ppm) were observed for all derivatives, corresponding to protons of the *para*-substituted benzene rings. The presence of these signals further confirms the incorporation of aromatic moieties into the chitosan polymeric structure.

On the other hand, the electronic nature of the substituents influences the chemical shift of the azomethine proton. Electron-donating groups such as –OH and –OCH_3_ exhibited relatively lower chemical shifts (~9.77–9.86 ppm), while electron-withdrawing groups such as –CO_2_Me, –CN, and –NO_2_ induced a progressive downfield shift up to ~10.20 ppm. This trend illustrates the deshielding effect that electron-withdrawing substituents exert on the imine functional group. Additionally, we observed characteristic signals associated with specific substituents. For example, the methoxy-substituted derivative (**3i**) showed a signal at 4.02 ppm, assigned to the –OCH_3_ group, while **3f** showed a signal at 3.42 ppm, attributed to the dimethylamine group. Although the experimental conditions were not optimized for quantitative analysis, the ^1^H NMR spectra provide clear qualitative evidence supporting the formation of Schiff base derivatives. These results are consistent with the structural assignments made using vibrational spectroscopy.

#### 3.6.3. Thermogravimetric Analysis (TGA) and Differential Scanning Calorimetry (DSC)

**Figure 2:**
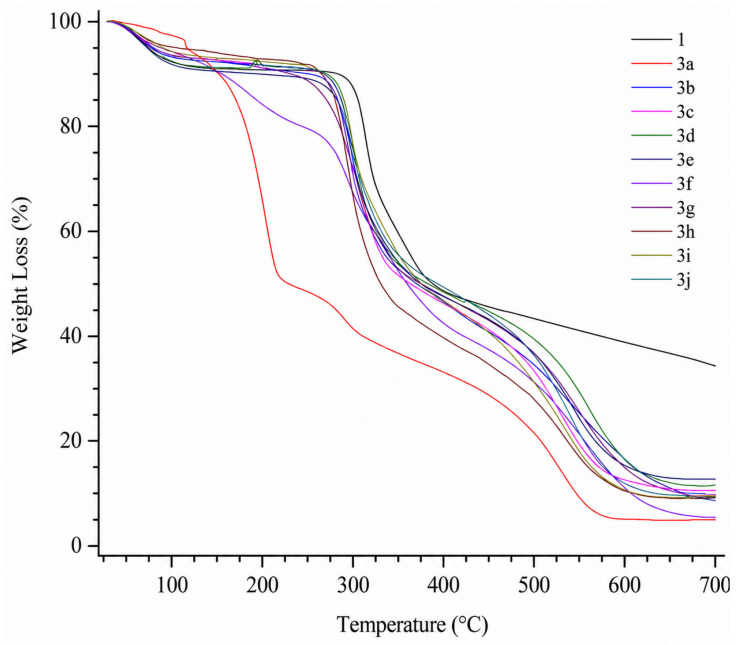
Thermogravimetric analysis (TGA) of chitosan **1** and Schiff bases **3a–j**.

Thermal properties of products made from chitosan were investigated. The TGA curves obtained for all Schiff bases (Figure 2), as observed in **1**, show an endothermic process below 100 °C, which is associated with the loss of water molecules that interact weakly via hydrogen bonds. The initial mass loss observed below 120 °C is attributed to the desorption of physically adsorbed water. The second stage of degradation is associated with cleavage of Schiff base linkages and with partial depolymerization. At higher temperatures, the major mass loss corresponds to the decomposition of the chitosan backbone and carbonization processes.

Compound **3a** exhibits significantly earlier degradation compared to the other Schiff bases. At 200 °C, 34% of its mass has already been lost, and by 300 °C, more than 50% has degraded. Similarly, compound **3f** exhibits lower thermal stability, although it is not as pronounced as that of **3a**. The reduced thermal stability of **3a** may be attributed to the hydroxyl group on the aromatic ring, which can form intramolecular and intermolecular hydrogen bonds, resulting in a less rigid structure more susceptible to thermal degradation. In contrast, the thermal stability difference between **3f** and **3a** is less pronounced and may be due to the strong electron-donating effect of the NMe_2_ group, which could destabilize the imine bond and consequently affect the thermal resistance of the biopolymer. Furthermore, chitosan exhibits greater thermal stability above 600 °C than Schiff bases, indicating a higher formation of stable carbonaceous residues in the unmodified biopolymer [45].

**Figure 3:**
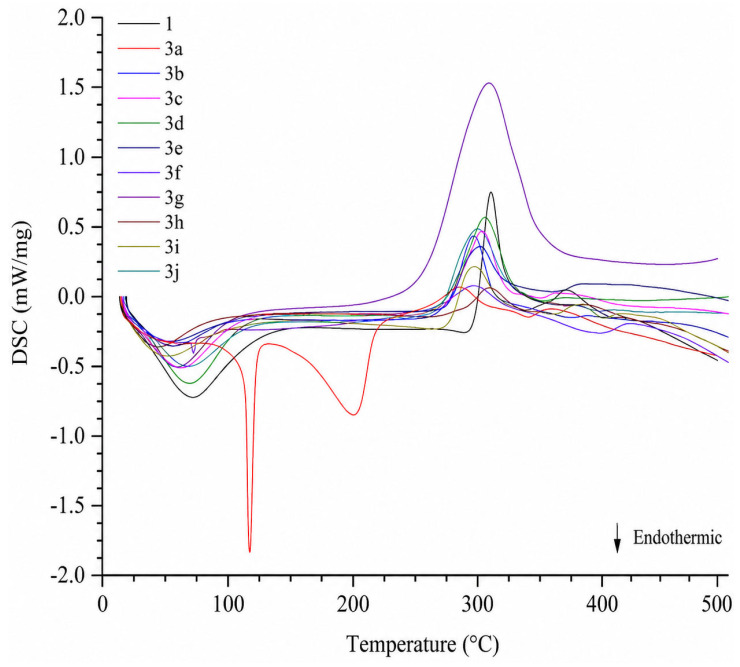
DSC curves of chitosan **1** and Schiff bases **3a–j**.

All Schiff bases display a similar DSC pattern to compound **1**, featuring an endothermic peak related to the loss of reticular water occurring at temperatures below 100 °C (Figure 3). This broad and less pronounced peak suggests a gradual thermal transition. However, in compound **3f**, a sharp overlapping peak is observed at 66 °C, which may indicate the melting point of the biopolymer. In contrast, compound **3a** exhibits a narrow, sharp peak at 111 °C, suggesting that this biopolymer has a higher melting point due to the presence of hydrogen bonds.

#### 3.6.4. Elemental Analysis

Table 5 shows the experimental results of the elemental analysis performed on the extracted chitosan **1**, commercial chitosan Qc and the synthesized Schiff’s base **3a**–**j**. The values obtained for **1** are similar to those of commercial chitosan. On the other hand, the results obtained for compounds **3a**–**j** show changes and differences in the percentages of nitrogen, carbon, hydrogen, and sulfur. These differences, in relation to the chitosan, are produced by the modification made to the biopolymer and highlight that the extracted chitosan was effectively functionalized.

#### 3.6.5. Cytotoxicity and Leishmania Activity

In the evaluation of leishmanicidal activity, only chitosan **1** and Schiff bases **3a** and **3i** show some activity against promastigotes of *L. mexicana*, with viability percentages of 47.3, 46.2, and 51.1, respectively, at 50 µg/mL (Table 6). In general, the presence of Schiff bases in the chitosan structure reduces its effect against parasites, except for compound **3a**, which has a hydroxyl group in the *ortho* position and retains activity. However, when the influence of substituents on activity is examined, compounds with electron-withdrawing substituents have lower activity than those with electron-donating groups. On the other hand, at a concentration of 100 µM, none of the compounds exhibit any cytotoxicity against RAW cells (Table 6), with compounds **3d** and **3g** being the most cytotoxic, with inhibition percentages of 72.7 and 76.6, respectively. A slight tendency suggests that chitosan derivatives with electron-withdrawing groups are more cytotoxic than those with electron-donating groups.

### 3.7. Characterization of Films

#### 3.7.1. AFM

All films were examined using atomic force microscopy, and the obtained micrographs are displayed in Figure S5 (Supplementary Materials). Table 7 shows the roughness values of all films. The roughness values for **3d**-PVA and PVA films are lower (1.630 nm and 1.243 nm, respectively) than those of plastic (1.995 nm). The low-density polyethylene (LDPE) surface has a low surface roughness (25.65 nm); in this sense, the **3d**-PVA, PVA, and the packaging plastic have a more homogeneous topography than the rest of the films studied [46]. The roughness of plastics treated to microbial deterioration shows topographies that increase following treatment [47].

Conversely, the roughness levels of the films fall within the nanometric range. The measured roughness values range from approximately 1 to 2 nm, indicating a similar topographic behavior among the produced films. These values are comparable to those obtained for PVA and packaging plastic, suggesting that all materials exhibit relatively smooth and homogeneous surfaces at the nanoscale. Therefore, differences in film performance are more likely associated with chemical composition and polymer chain interactions rather than surface roughness alone. Additionally, chitosan tends to form aggregates due to intermolecular and intramolecular hydrogen bonding, which promotes a compact macrostructure and contributes to the observed surface smoothness [48].

#### 3.7.2. CO_2_/O_2_ Concentration Using Films Based on Schiff Base with PVA as Simulation Packing Material

Films 1–PVA, 3a–PVA, and 3b–PVA were selected for permeability studies based on their combined biological activity, film-forming ability, structural stability, and representative electronic effects of the para-substituents. Schiff bases **3a** and **3b** were selected to evaluate their potential as food packaging materials. The results are shown in Table 8 and Figure S6. Minimally processed carrots packaged with films **1**–PVA, **3a**–PVA, and **3b**–PVA showed a decrease in O_2_ concentration and an increase in CO_2_ production (Table 8), consistent with the natural respiration process of the vegetable. This passive modified atmosphere contributes to shelf-life extension by reducing the respiration rate [49,50,51,52,53]. Film **1**–PVA exhibited greater CO_2_ accumulation than **3a**–PVA and **3b**–PVA. In contrast, **3a**–PVA maintained a higher O_2_ concentration compared to **1**–PVA and **3b**–PVA. These results suggest that **1**–PVA and **3b**–PVA are more permeable to O_2_, while **3a**–PVA limits CO_2_ exchange. Additionally, **1**–PVA retained CO_2_ more effectively, whereas **3b**–PVA and **3a**–PVA, which showed statistically similar CO_2_ values, displayed comparable O_2_ behavior and higher permeability to CO_2_.

Chitosan is a widely studied biomaterial for the development of films and coatings for food applications [49,50,51,52], as it has been shown to reduce the respiration rate of vegetables [49,51]. The results obtained indicate that chitosan functionalization affects CO_2_/O_2_ exchange, most likely through changes in chemical composition and polymer chain interactions rather than surface roughness alone, since all films exhibited comparable nanometric roughness values (Section 3.7.1). This suggests that gas selectivity could be influenced by intermolecular interactions between CO_2_ and O_2_ on the film surface. Additionally, the composition of the synthesized film plays a significant role in its gas exchange properties [53].

The structural and barrier properties of films prepared from functionalized chitosan combined with polyvinyl alcohol (PVA) were evaluated. The incorporation of functionalized derivatives altered surface roughness and gas permeability. In particular, **1**–PVA films exhibited greater CO_2_ retention compared to **3a**–PVA and **3b**–PVA, whereas 3a–PVA maintained higher O_2_ concentrations in trials with minimally processed carrots. Further studies are warranted to determine the respiration rate and the permeability to water vapor, ethylene, CO_2_, and O_2_ to evaluate the potential of these films as eco-friendly packaging alternatives. The chitosan films enriched with bioactive compounds enhance gas barrier properties and contribute to food preservation through the generation of passive modified atmospheres.

Similarly, atomic force microscopy (AFM) revealed that all films exhibited nanometric roughness values. Therefore, selective gas transmission is more likely associated with chemical composition, intermolecular interactions, and polymer chain mobility than with surface roughness alone. These observations suggest that surface topography, together with chemical composition and polymer chain interactions, may contribute to selective gas transmission in these films.

#### 3.7.3. Mechanical Properties of Films

The mechanical properties of the films were evaluated to assess their structural performance and potential applicability in packaging materials (Table S3 and Figure S19). Tensile tests were performed using a universal testing machine (Shimadzu), and the results are summarized in Table 9.

The results reveal that the incorporation of Schiff base derivatives into the chitosan–PVA matrix significantly influences both tensile strength and elongation behavior, indicating that chemical functionalization plays a key role in tuning the mechanical performance of the films. In this sense, the film corresponding to **3a–PVA** (–OH substituent) exhibited a high tensile strength (~892 N/mm^2^) combined with considerable elongation (~396%), suggesting an optimal balance between rigidity and flexibility. This behavior can be attributed to the presence of hydroxyl groups, which promote intermolecular hydrogen bonding, leading to improved structural cohesion. This observation is consistent with DSC results, where **3a** showed a sharper thermal transition, indicative of a more organized structure. In contrast, **3g–PVA** (–NO_2_ substituent) displayed the highest elongation (~555%), indicating enhanced flexibility. The electron-withdrawing nature of the nitro group may reduce intermolecular interactions between polymer chains, resulting in increased chain mobility. This agrees with TGA/DSC results, where modified derivatives generally showed reduced thermal stability compared to pure chitosan, suggesting less rigid polymer networks. Similarly, derivatives such as **3e–PVA** (–F) and **3d–PVA** (–CN) exhibited intermediate mechanical behavior, with moderate tensile strength and high elongation values. These substituents introduce polarity into the system, which may influence intermolecular interactions and contribute to a balance between stiffness and flexibility. Some variability between replicates was observed, particularly for **3f–PVA**, which may reflect structural heterogeneity of the films and differences in intermolecular interactions within the polymeric matrix.

Because some films were tested in duplicate or as single measurements due to material availability, the mechanical results should be interpreted as preliminary comparative data. Future work should include at least three independent replicates per formulation. The **3f–PVA** (–NMe_2_) film showed high variability in tensile strength, indicating heterogeneity in the film structure. This behavior may be associated with the strong electron-donating nature of the dimethylamino group, which can affect imine stability, as previously suggested in the thermal analysis section. Compared to the reference material (**PVA** alone), which exhibited relatively low elongation (~65%), most modified films demonstrated significantly improved flexibility, highlighting the role of chitosan functionalization in enhancing mechanical performance. From a structural perspective, the mechanical properties correlate with AFM observations (Section 3.7.1). Films with low roughness values (1–2 nm) exhibited relatively homogeneous mechanical behavior, whereas differences in mechanical performance are more strongly associated with chemical composition rather than surface morphology alone. Furthermore, the relationship between mechanical properties and gas permeability (Section 3.7.2) suggests that films with higher flexibility, such as **3g–PVA**, may facilitate greater gas diffusion due to increased polymer chain mobility, while more rigid structures such as **3a–PVA** may contribute to more controlled gas exchange. Overall, these results demonstrate that the nature of the substituent in Schiff base-modified chitosan directly affects the mechanical behavior of the resulting films. This tunability is particularly relevant for the design of biodegradable packaging materials, where an appropriate balance between strength, flexibility, and permeability is required.

## 4. Conclusions

Chitosan was successfully obtained from shrimp shell waste and subsequently functionalized with para-substituted benzaldehydes to synthesize Schiff base–chitosan derivatives. The chitosan obtained from shrimp shells evidenced a molecular weight of 1521.5 kDa. The deacetylation degree determined by potentiometric titration and elemental analysis showed 53.4% and 68.6%. Furthermore, the deacetylation degree observed by infrared and Raman spectroscopy evidenced values of 71.8% and 75.6%. In addition, Schiff base-chitosan derivatives were characterized using infrared and Raman spectroscopy, thermogravimetric analysis (TGA), and elemental analysis. The structural characterization of the synthesized derivatives was supported by complementary spectroscopic techniques. In addition to IR and Raman analyses, ^1^H NMR spectroscopy provided clear qualitative evidence of Schiff base formation through the appearance of characteristic azomethine signals and aromatic resonances. These results confirm the successful functionalization of chitosan and the preservation of its polymeric backbone. TGA studies revealed that the presence of benzene and imine groups, as well as substituents in the ring’s *para* position, affects the products’ thermal degradation.

In vitro biological experiments demonstrated that chitosan **1** and Schiff bases **3a** and **3i** exhibit slight leishmanicidal activity against promastigotes of *Leishmania mexicana* at a concentration of 50 µg/mL. Furthermore, when the concentration was doubled, the derivatives showed no significant cytotoxicity in RAW cell culture. Films made from some functionalized derivatives that integrate PVA as a plasticizer were examined for their potential use as packaging materials. These films displayed barrier qualities that affected the respiration rate of minimally processed carrots. Test on the CO_2_ and O_2_ respiration rates in a confined and static environment using **1**-PVA films showed more CO_2_ accumulation than **3a**-PVA and **3b**-PVA films.

Taken together, this study confirms that high-molecular-weight chitosan extracted from shrimp waste can be effectively functionalized to obtain derivatives with both biological and packaging-related functional properties. The synthesized biopolymers not only exhibit potential for antiparasitic applications but also show promising features for the development of eco-friendly packaging materials with controlled gas permeability. Future research could focus on the incorporation of nitrogen-containing heterocycles or other bioactive groups to enhance antiparasitic efficacy, as well as on the exploration of synergistic blends with other biopolymers to optimize mechanical properties and gas transmission selectivity. Finally, mechanical characterization showed that Schiff base modification enables tuning of film properties, with variations in tensile strength and flexibility depending on the substituent. Together with thermal and surface analyses, these results support the potential of these materials for biodegradable packaging applications.

## Figures and Tables

**Table 1:** Mass of chitosan and aldehyde used for the synthesis of each polymer product.

Chitosan Mass (mg)	R	Mass of Benzaldehyde (mg)	Schiff Base
400.1	OH	304.2	**3a**
500.4	COOMe	511.4	**3b**
400.2	Br	460.9	**3c**
400.3	CN	326.8	**3d**
400.1	F	309.1	**3e**
400.1	NMe_2_	371.6	**3f**
400.3	NO_2_	376.6	**3g**
500.6	OCF_3_	592.5	**3h**
400.1	OCH_3_	339.1	**3i**
500.5	SCH_3_	474.2	**3j**

**Table 2:** Different viscosities for chitosan **1** solutions at 20 °C, calculated using the Oswald viscometer.

Concentration (mg/mL)	Time (s) ^a^	*ɳ*_r_*	*ɳ*_sp_*	ɳ*_red_* (mL/g)
0 ^b^	1.73 ± 0.13	---	---	---
2	2.49 ± 0.05	2.609	1.609	805
4	3.62 ± 0.09	4.602	3.602	901
6	8.41 ± 0.12	12.158	11.158	1860
8	22.94 ± 0.35	33.946	32.946	4118
10	49.71 ± 2.65	74.315	73.315	7332

^a^: average of four measurements; ^b^ 1% acetic acid; ɳ*_r_* = dynamic viscosity; ɳ*_sp_* = specific viscosity; ɳ*_red_* = reduced viscosity * dimensionless.

**Table 3:** Degree of Deacetylation of chitosan **1**.

Degree of Deacetylation (%)			
IR ^a^	Raman ^b^	P ^c^	EA ^d^
71.8	75.6	53.4	68.6

^a^: IR spectroscopy, ^b^: Raman spectroscopy, ^c^: Potentiometric titration, ^d^: Elemental analysis.

**Table 4:** Principal vibrational modes of functionalized chitosan **3a–j**.

Compound	IR (cm^−1^)	Raman (cm^−1^)	Assignment
**3a**	1645 sh (m)	1662 (m)	ν (C=N) imine
	1218 (s)	1213 (w)	ν (OH)
**3b**	1719 (s)	1716 (w)	ν (C=O) ester
	1646 (s)	1638 (s)	ν (C=N) imine
	1645 (s)	1640 (m)	ν (C-O) ester
	1287 (s)	1220 (m)	ν (OH)
**3c**	1645 (s)	1644 (m)	ν (C=N) imine
	1071 (s)	---	ν (C-Br) aromatic
**3d**	2229 (m)	2231 (m)	ν (CN)
	1646 (s)	1644 (m)	ν (C=N) imine
**3e**	1646 (s)	1638 (m)	ν (C=N) imine
	1232 (s)	1231 (m)	ν (C-F) aromatic
**3f**	1659 (m)	1638 (m)	ν (C=N) imine
	1232 (w)	1247 (w)	δ (C-N-C)
**3g**	1646 (s)	1656 (m)	ν (C=N) imine
	1523 (s)	---	δ (NO_2_)
**3h**	1649 (s)	1641 (m)	ν (C=N) imine
	1261 (s)	1223 (m)	ν (CF_3_)
**3i**	1652 (m)	1638 (m)	ν (C=N) imine
	1255 (s)	1226 (m)	δ (C-O-C)
**3j**	1642 (s)	1641 (m)	ν (C=N) imine
	1435 (w)	1452 (w)	δ (C-S-C)

ν: stretching; δ: deformation; sh: Shoulder-shaped band; s: strong; m: medium; w: weak.

**Table 5:** Carbon, nitrogen, hydrogen, and sulfur content of chitosan and functionalized chitosans.

Compound	N [%]	C [%]	H [%]	S [%]
**1**	6.91	39.27	7.88	---
**3a**	5.89	43.73	6.60	---
**3c**	4.61	41.41	5.42	---
**3d**	7.62	47.40	5.68	---
**3e**	4.85	42.54	6.01	---
**3f**	6.94	48.31	6.77	---
**3g**	7.37	43.91	5.33	---
**3h**	4.51	42.93	5.11	---
**3i**	5.33	44.64	6.54	---
**3j**	4.97	45.21	5.96	4.27
Qc	6.95	39.89	7.43	---

**Table 6:** Viability percentages of chitosan and synthesized products against promastigotes of *L. mexicana* and RAW 264.7 cells.

	*Leishmania mexicana* ^a^	RAW 264.7 ^b^
Acetic acid 2%.	121.6 ± 20.4	95.4 ± 2.3
**1**	47.3 ± 2.8	92.2 ± 7.4
**3a**	46.2 ± 3.2	91.6 ± 7.1
**3b**	90.6 ± 5.1	82.4 ± 4.1
**3c**	102.5 ± 13.7	87.5 ± 6.9
**3d**	100.8 ± 6.3	72.7 ± 6.7
**3e**	91.1 ± 10.3	86.4 ± 5.1
**3f**	78.9 ± 0.4	84.1 ± 6.1
**3g**	107.0 ± 14.0	76.6 ± 9.0
**3h**	72.9 ± 0.7	81.7 ± 5.1
**3i**	51.1 ± 4.8	83.4 ± 7.4
**3j**	85.4 ± 9.9	88.9 ± 5.5
Amphotericin B	15.1 ± 3.6	---
Saponine ^c^	---	3.1 ± 0.4

^a^: 50 µg/mL, ^b^: 100 µg/mL, ^c^: 2.4 mg/mL.

**Table 7:** Roughness of the functionalized chitosan films.

Film	Roughness (nm)
**1**–PVA	1.0
**3a**–PVA	1.0
**3b**–PVA	2.0
**3c**–PVA	2.0
**3d**–PVA	1.63
**3e**–PVA	1.0
**3f**–PVA	2.0
**3g**–PVA	2
**3h**–PVA	1
**3i**–PVA	1
**3j**–PVA	1
Qc–PVA	1
PVA	1.24
Plastic of packaging	1.99

Film made with 50%PVA and 50% chitosan or functionalized chitosan.

**Table 8:** Headspace gas composition of packaged samples.

Film	O_2_ (%)	CO_2_ (%)
**1**–PVA	16.7 ± 0.6	6.8 ± 1.9
**3a**–PVA	17.7 ± 0.2	4.1 ± 0.8
**3b**–PVA	16.4 ± 0.2	4.8 ± 0.6

**Table 9:** Mechanical properties of chitosan–Schiff base/PVA films.

Film	R	Tensile Strength (N/mm^2^)	Elongation (%)	Displacement (mm)
	(*Para*-Substituted Benzaldehyde)			
**1–PVA**	—	265.35 *	64.74 *	5.18 *
**3a–PVA**	–OH	892.75 ± 23.42	395.85 ± 36.67	31.67 ± 2.93
**3b–PVA**	–COOMe	146.07 ± 3.43	395.45 ± 47.24	31.64 ± 3.78
**3c** **–** **PVA**	–Br	66.94 ± 5.85	334.90 ± 101.56	26.79 ± 8.12
**3d–PVA**	–CN	194.05 ± 14.21	454.58 ± 1.83	36.37 ± 0.15
**3e–PVA**	–F	225.84 *	441.46 *	35.32 *
**3f–PVA**	–NMe_2_	1053.68 ± 659.36	267.36 ± 157.63	21.39 ± 12.61
**3g–PVA**	–NO_2_	123.33 ± 19.87	555.17 ± 23.56	44.41 ± 1.89
**3i–PVA**	–OCH_3_	161.72 ± 36.24	490.30 ± 164.73	39.22 ± 13.18
**Qc–PVA**	—	308.70 ± 67.08	514.65 ± 80.57	41.17 ± 6.45

Values are expressed as mean ± standard deviation (n = 2). * Single measurement (n = 1). Films were prepared using 50% (*w*/*w*) PVA and 50% chitosan or functionalized chitosan derivative. Tensile properties were measured using a Shimadzu universal testing machine under controlled conditions at room temperature. Tensile strength corresponds to maximum stress at break; elongation represents strain at break. The R substituent corresponds to the *para*-substituted benzaldehyde used for Schiff base formation (see Scheme 1).

## Data Availability

The original contributions presented in this study are included in the article/supplementary material. Further inquiries can be directed to the corresponding author.

## Supplementary Materials

The following supporting information can be downloaded at https://www.mdpi.com/article/10.3390/polym18111397/s1, Figure S1: FT–IR and Raman spectra of chitosan; Figure S2: Chitosan titration curve; Figure S3: DSC (a)/TGA (b) of chitosan; Figure S4: DSC/TGA of the synthesized products; Figure S5: AFM plots of the fabricated films. (a) 1–PVA, (b) 3a–PVA, (c) 3b–PVA, (d) 3c–PVA, (e) 3d–PVA, (f) 3e–PVA, (g) 3f–PVA, (h) 3g–PVA, (i) 3h–PVA, (j) 3i–PVA. (k) 3j–PVA, (l) Qc–PVA, (m) PVA, (n) Plastic for packaging; Figure S6: Gas kinetics graphs. (a) O2, (b) CO2; Figure S7: Plot of reduced viscosity vs chitosan concentration; Figure S8: O2 concentration in jars with carrots packed with films of chitosan 1-PVA and Schiff bases 3a–PVA, 3b-PVA; Figure S9: CO2 concentration in jars with carrots packed with films of chitosan 1-PVA and Schiff bases 3a–PVA, 3b-PVA; Figure S10: 1H-NMR spectra of chitosan 1; Figure S11: 1H-NMR spectra of 3a; Figure S12: 1H-NMR spectra of 3b; Figure S13: 1H-NMR spectra of 3c; Figure S14: 1H-NMR spectra of 3d; Figure S15: 1H-NMR spectra of 3e; Figure S16: 1H-NMR spectra of 3f; Figure S17: 1H-NMR spectra of 3g; Figure S18: 1H-NMR spectra of 3i; Figure S19: Experimental stress–strain representation of selected films (1–PVA, 3a–PVA, 3g–PVA, and Qc–PVA) based on individual replicate data. Each line corresponds to a single measurement using maximum tensile strength and elongation at break values. Table S1: Fundamental vibrational modes of chitosan **1**; Table S2: Carbon, nitrogen, hydrogen and sulfur content in chitosan **1**; Table S3: Raw mechanical data of chitosan–Schiff base/PVA films.
