# Supplementary material for: High Molecular Weight Chitosan from Shrimp Shells: Synthesis of Para-Substituted Schiff Bases with Selective Leishmanicidal Activity and Application in CO2/O2-Selective Films
Source: Polymers (Basel). 2026 Jun 4;18(11):1397. doi: 10.3390/polym18111397 (PMC13259417; doi:10.3390/polym18111397)
Supplement: Supplementary file 1 [file polymers-18-01397-s001.zip › polymers-4195933-supplementary.pdf]

Supplementary Material

High Molecular Weight Chitosan from Shrimp Shells: Synthesis of *para*-Substituted Schiff Bases with Selective Leishmanicidal Activity and Application in CO_2/O_2-Selective Films

Andrés Alejandro Yánez-Crespo [1], Christian David Alcívar-León [1,*], Pablo Mauricio Bonilla-Valladares [1], Trosky Germán Yánez-Darquea [1], Jorge Heredia-Moya [2,*], Luciana Juncal [3], A. Fabiana Cabrera [3], María José Andrade-Cuvi [4], Carlota Moreno-Guerrero [5], Sonia E. Ulic [6]

1 Facultad de Ciencias Químicas, Universidad Central del Ecuador, Francisco Viteri y Gilberto Sobral s/n, Ciudad Universitaria, Quito, Ecuador.
2 Centro de Investigación Biomédica (CENBIO), Facultad de Ciencias de la Salud Eugenio Espejo, Universidad UTE, Quito 170527, Ecuador.
3 Instituto de Física La Plata (IFLP), CONICET, Departamento de Física, Facultad de Ciencias Exactas, Universidad Nacional de La Plata, Diagonal 113 e/ 63 y 64, 1900, La Plata, Argentina.
4 Laboratorio de Investigación en Ingeniería en Alimentos (LabInAli), Departamento de Ingeniería en Alimentos, Colegio de Ciencias e Ingenierías, Universidad San Francisco de Quito USFQ, Quito 170901, Ecuador.
5 Centro de Investigación de Alimentos, CIAL, Facultad de Ciencias de la Ingeniería e Industrias, Universidad UTE, EC171029 Quito, Ecuador.
6 CEQUINOR (UNLP-CONICET), Universidad Nacional de La Plata, La Plata 1900, Argentina.

* Correspondence: cdalcivar@uce.edu.ec (C.D.A.L); jorgeh.heredia@ute.edu.ec (J.H.-M.).

Equations

Equation S1: Dynamic viscosity

$$\eta = k_1 \delta t - k_2 \frac{\delta}{t}$$

Where η is the dynamic viscosity of the solution, k_1 and k_2 are the viscometer's own constants, δ and t are the density and time to fall through the viscometer of each solution.

Equation S2: Relative viscosity

$$\eta_r = \frac{\eta}{\eta_0}$$

Where η_r is the relative viscosity, η is the dynamic viscosity which is calculated using the time and density data of each solution and η_0 is the dynamic viscosity of the pure solvent.

Equation S3: Specific viscosity

$$\eta_{sp} = \eta_r - 1$$

Equation S4: Reduced viscosity

$$\eta_{red} = \frac{\eta_{sp}}{C}$$

Where C is the concentration in grams per 100 mL of solution.

Example of stoichiometric calculation for synthesis

Stoichiometric calculation to determine the amount of aldehyde used in the reactions.

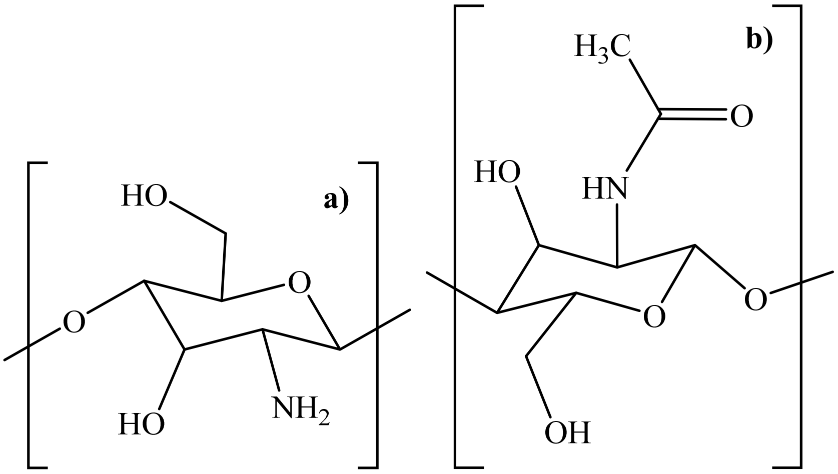

(a) β-(1-4) D-glucosamine (deacetylated units G), **(b)** N-acetyl-D-glucosamine (acetylated unit AC).

$$PM_G = 161.1558$$

$$PM_{AC} = 203.1925$$

$$PM_{Q1} = PM_G(71.8\ \%) + PM_{AC}(28.2\ \%)$$

$$PM_{Q1} = 161.1558 * 71.8\ \% + 203.1925 * 28.2\ \%$$

$$PM_{Q1} = 17301.0145\ g/mol$$

For a 1:1 Ratio

$1Q_1 + 71.8$ Aldehyde → Base de Schiff + H_2O

17301.0145 → 8768.216

1521467.919 → X

$$X = 771085.3806$$

1521467.919 → 771085.3806

0.4001 g → X

$$X = 0.202721\ g\ Aldehyde$$

For a 1:1,5 Ratio

0.202721 $g\ Aldehyde$ → 1 $mol\ de\ Aldehyde$

$Xg\ Aldehyde$ ← 1,5 $mol\ de\ Aldehyde$

$$Xg = 0.304158\ g$$

For the calculation, aldehyde **2a** (R-OH) was taken as an example.

Tables

Table S1. Fundamental vibrational modes of chitosan **1**.

**Experimental**		**Assignment***
**IR (cm^{-1})**	**Raman (cm^{-1})**	
3430	---	ν (OH)HB
3369	---	ν (OH)
3279sh	---	νas (NH_2)
3104sh	---	νs (NH_2)
2919	2933sh	ν (CH_3)
2878	2884	νas (CH_2)
2854	2867	νs (CH_2)
1653	1654	ν (CO) amide
1561	---	δ (NH_2)
1551	1552	δ (C-N-H) amide
1457	1456	δ (CH); (CH2); δ(OH)
1420	1420sh	δ (CH3)
1379	1378	δ (C-C-H); δ (CH_2); δ(OH)
1320	1321	ν (C-N); δ (-CH_2-)
1262	1263sh	δ (OH…O); ν (C-C); ν (C-O); δ (CH); γ (CH_2)
1154	1148	γ (CH_2); δ (CH_3)
1074	1075	νas (C-O) ether
1033	---	ν (ϕ); δ (CH)
952	---	ν (CN)
898	896	ν (ϕ); δ (CH_2)
562	---	γ (NH); γ (C=O)
---	424	γ (OH)

* ν, νas; stretching and asymmetric stretching. ϕ: Pyranose ring δ, γ; in-plane deformation and out-of-plane deformation. sh: Shoulder-shaped band. HB: Hydrogen bridge.

Table S2. Carbon, nitrogen, hydrogen and sulfur content in chitosan **1**.

Name	N [%]	C [%]	H [%]	S [%]
1	6,91	39,27	7,878	0,170

Figures

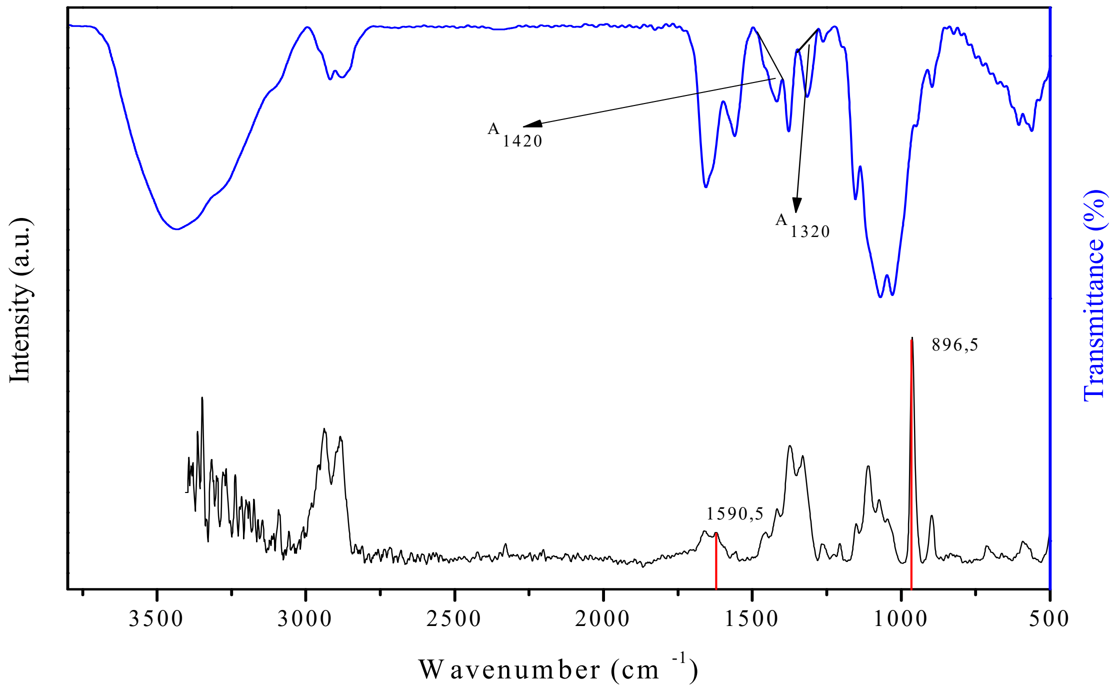

Figure S1. FT–IR and Raman spectra of chitosan.

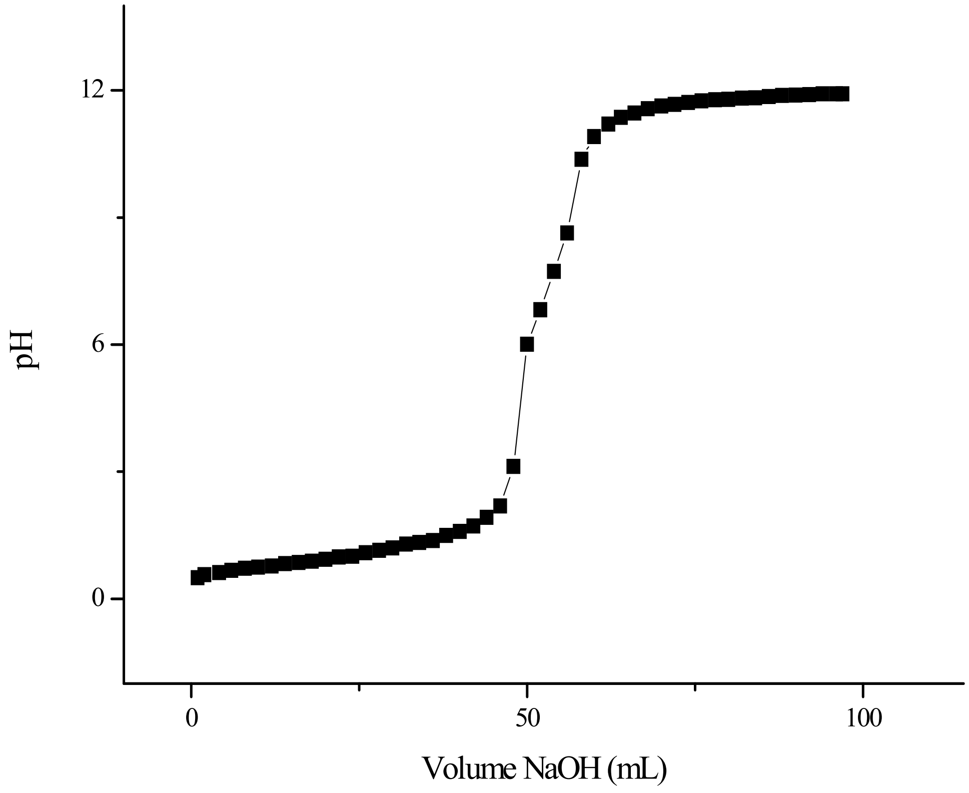

Figure S2. Chitosan titration curve.

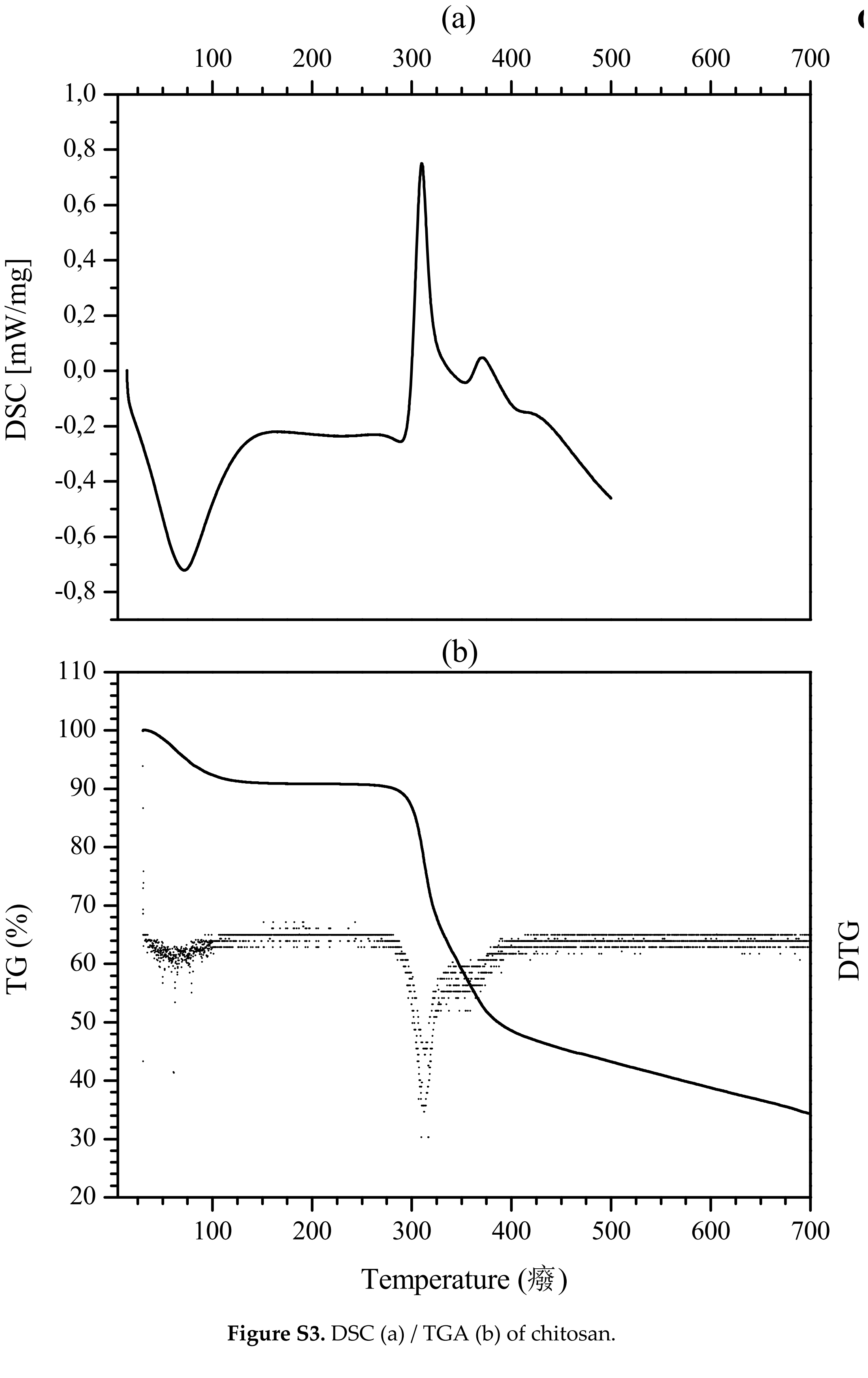

Figure S3. DSC (a) / TGA (b) of chitosan.

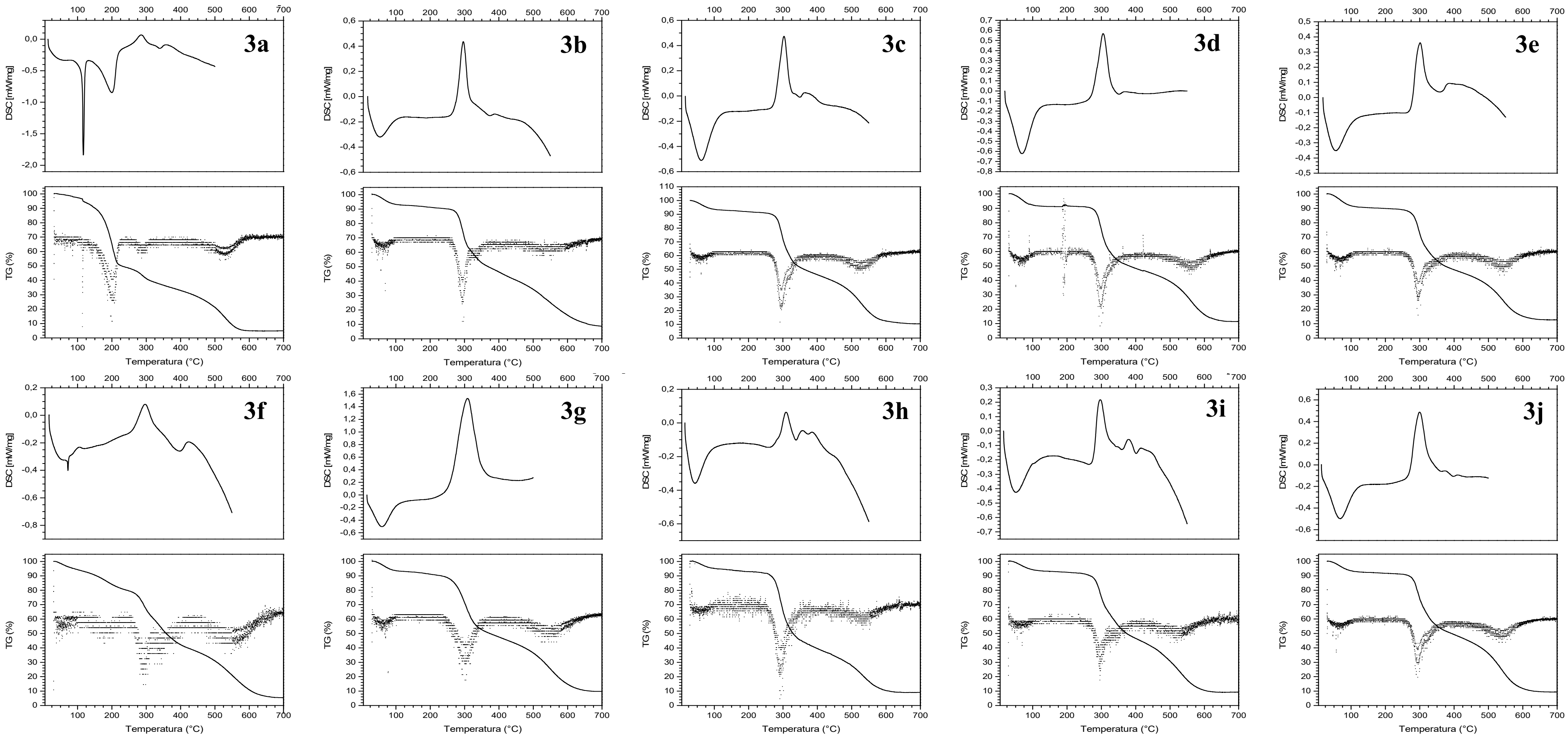

Figure S4. DSC / TGA of the synthesized products.

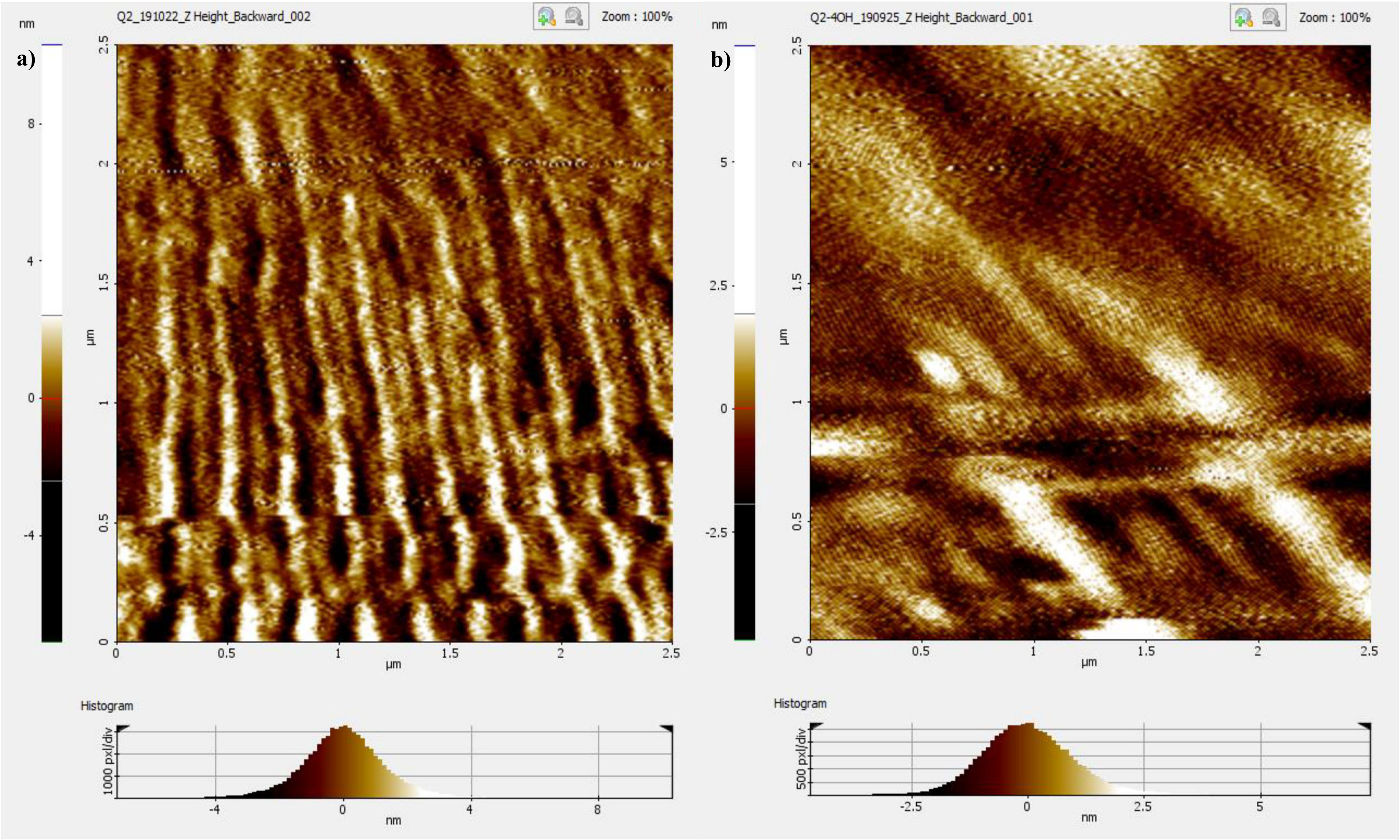
a)
Q2_191022_Z Height_Backward_002
Zoom : 100%
nm
µm
µm
Histogram
1000 pxl/div
nm
b)
Q2-4OH_190925_Z Height_Backward_001
Zoom : 100%
nm
µm
µm
Histogram
500 pxl/div
nm

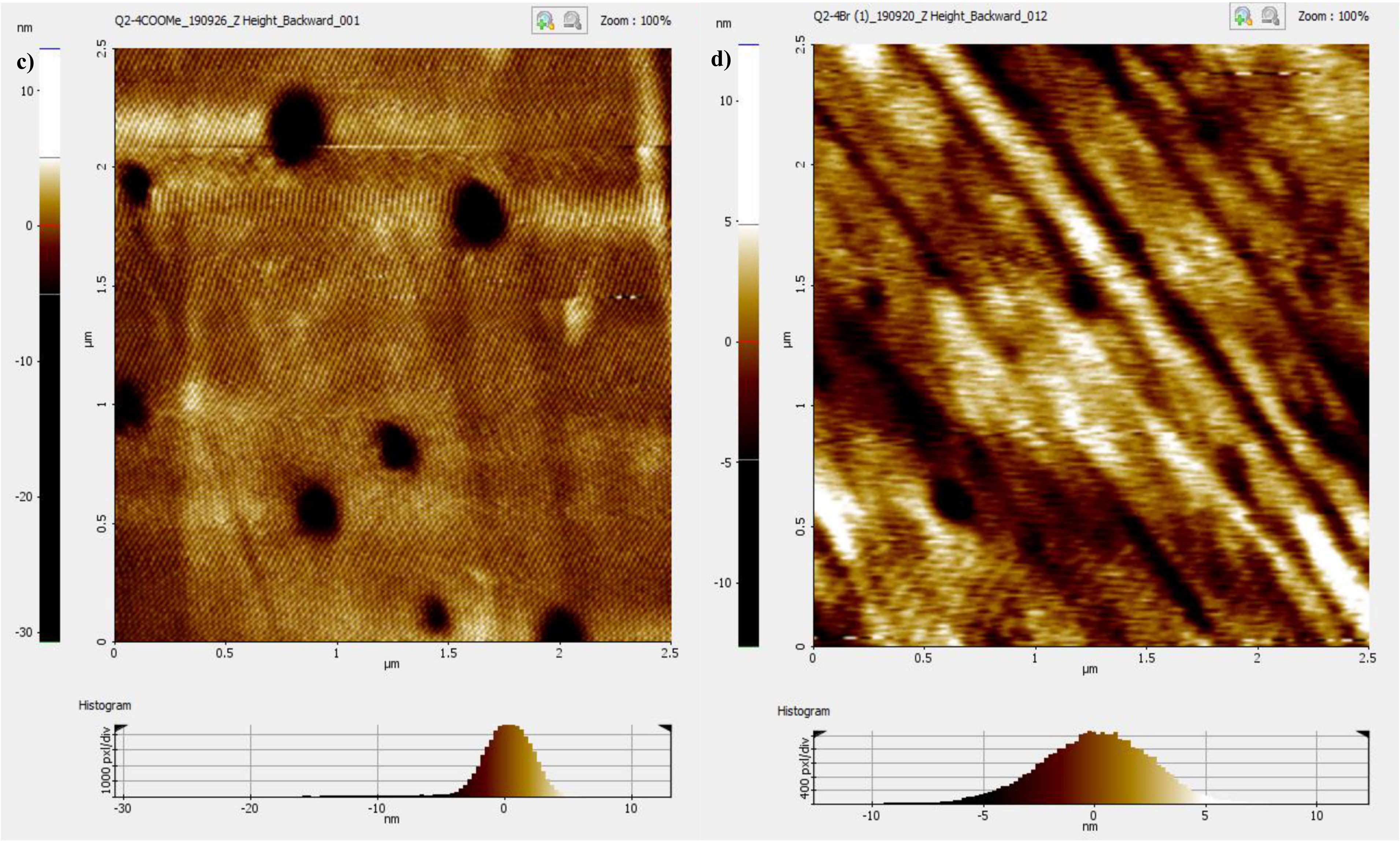
c)
Q2-4COOMe_190926_Z Height_Backward_001
Zoom : 100%
nm
µm
Histogram
1000 pxl/div
d)
Q2-4Br (1)_190920_Z Height_Backward_012
Zoom : 100%
nm
µm
Histogram
400 pxl/div

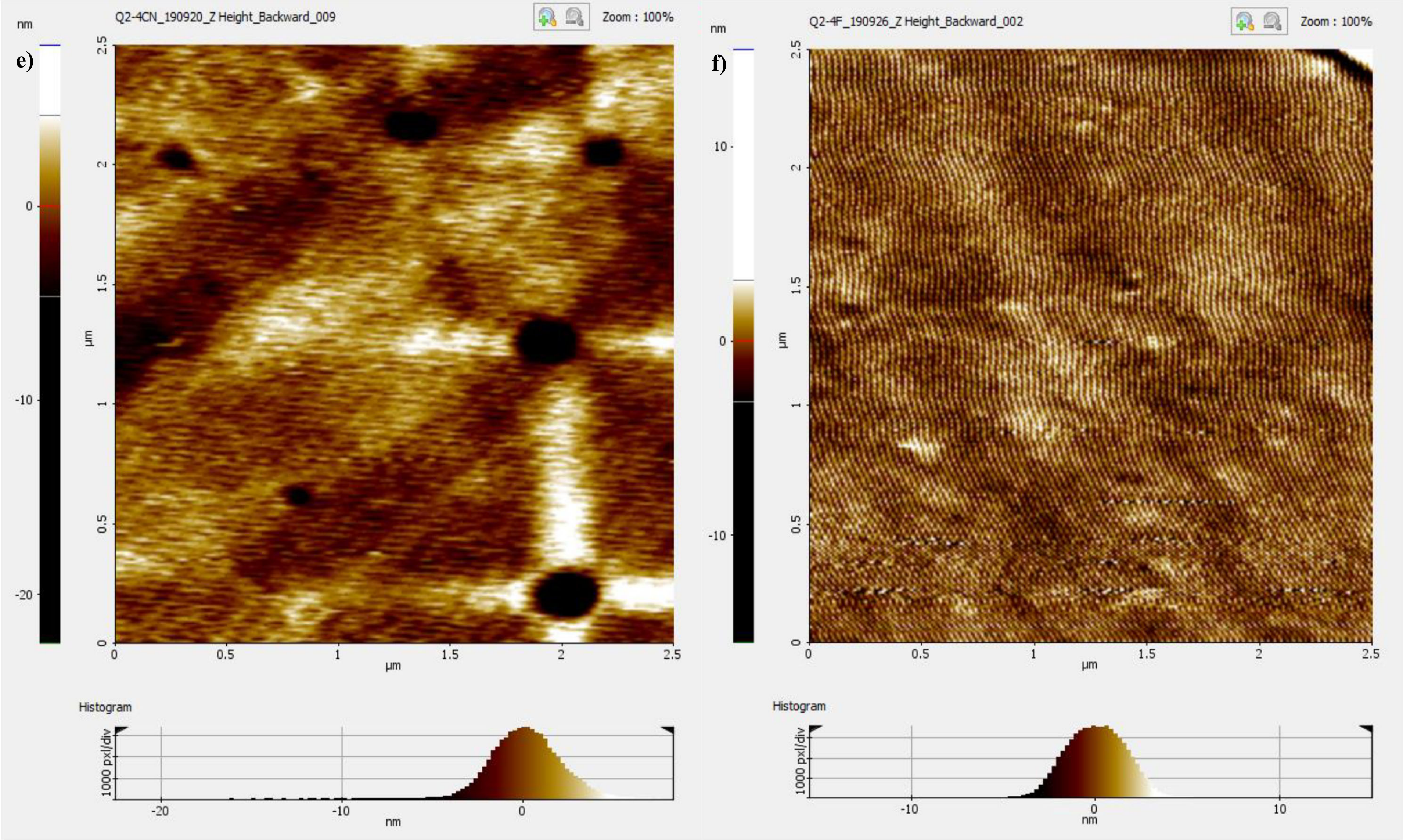

e)
Q2-4CN_190920_Z Height_Backward_009
Zoom : 100%
nm
0
-10
-20
μm
0
0.5
1
1.5
2
2.5
Histogram
1000 pxl/div
f)
Q2-4F_190926_Z Height_Backward_002
Zoom : 100%
nm
10
0
-10
μm
0
0.5
1
1.5
2
2.5
Histogram
1000 pxl/div

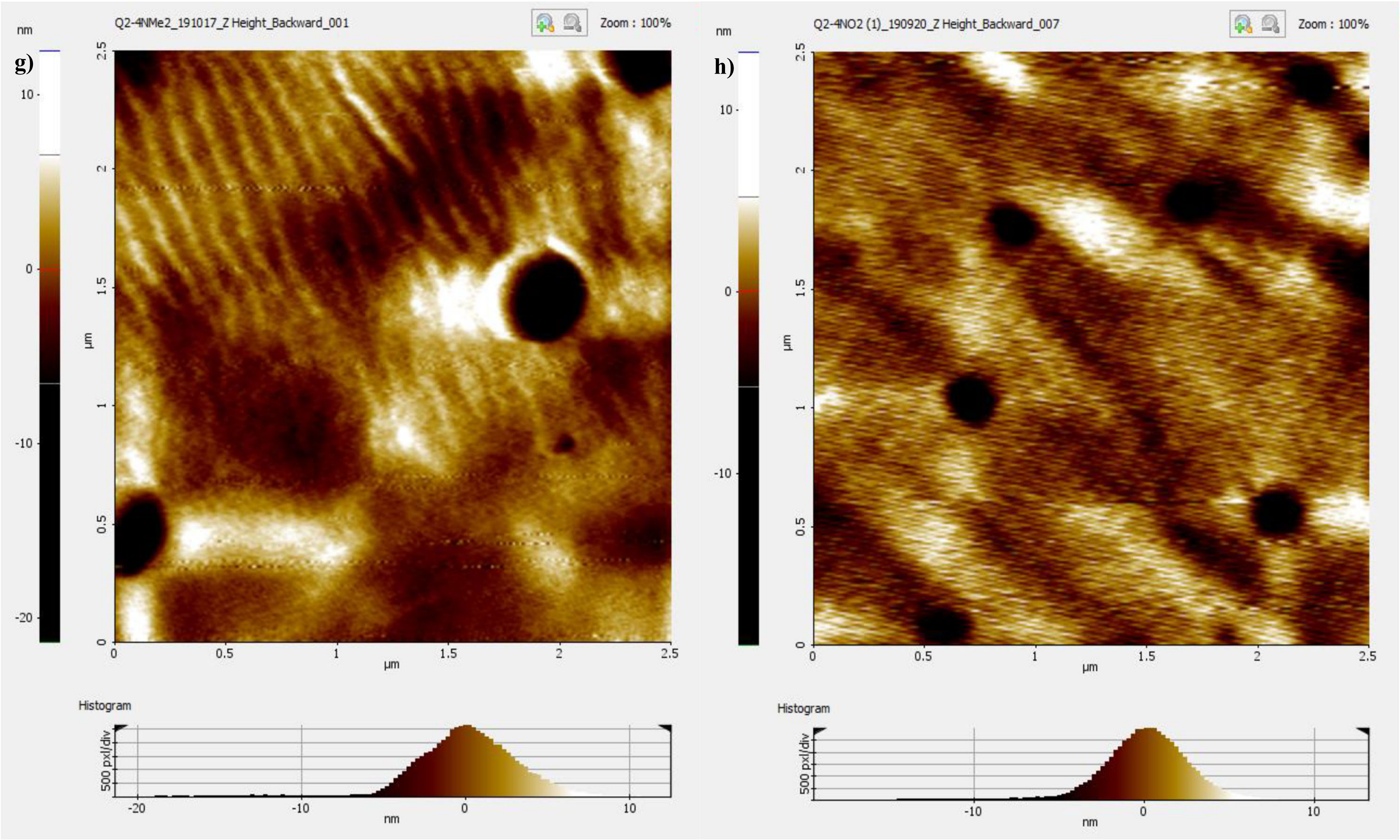
g)
Q2-4NMe2_191017_Z Height_Backward_001
Zoom : 100%
nm
μm
Histogram
500 pxl/div
h)
Q2-4NO2 (1)_190920_Z Height_Backward_007
Zoom : 100%
nm
μm
Histogram
500 pxl/div

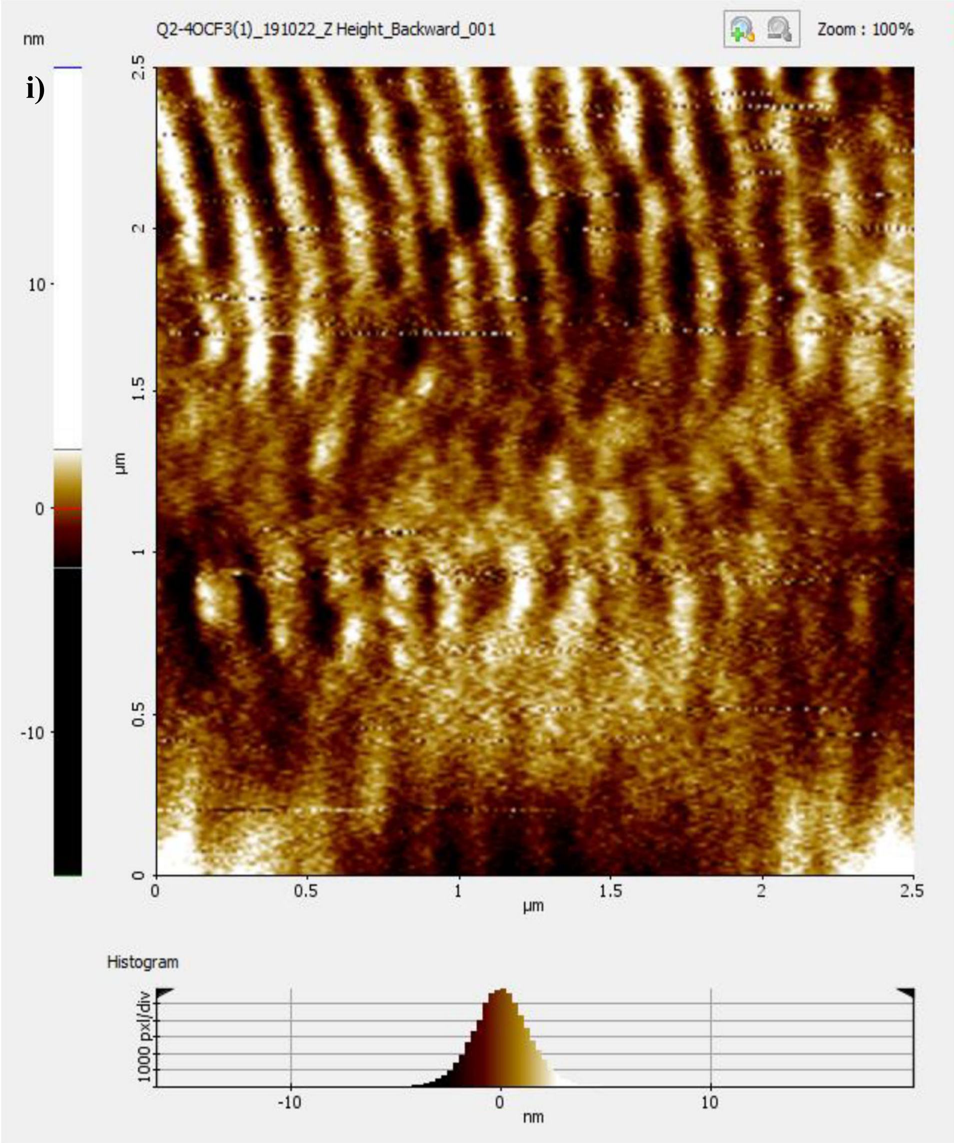
i)
Q2-4OCF3(1)_191022_Z Height_Backward_001
Zoom : 100%
nm
10
0
-10
µm
0
0.5
1
1.5
2
2.5
Histogram
1000 pxl/div
nm

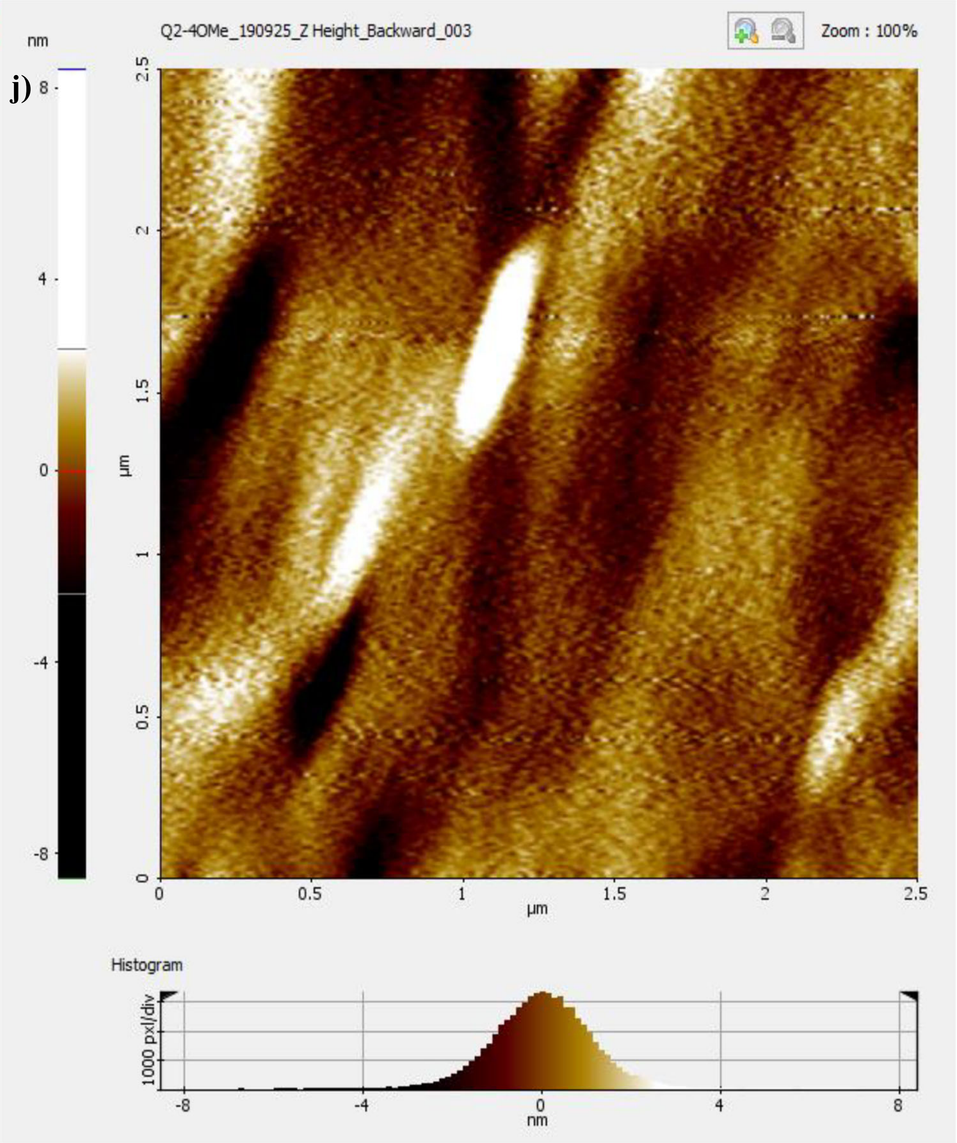
j)
Q2-4OMe_190925_Z Height_Backward_003
Zoom : 100%
nm
8
4
0
-4
-8
µm
0
0.5
1
1.5
2
2.5
Histogram
1000 pxl/div
nm

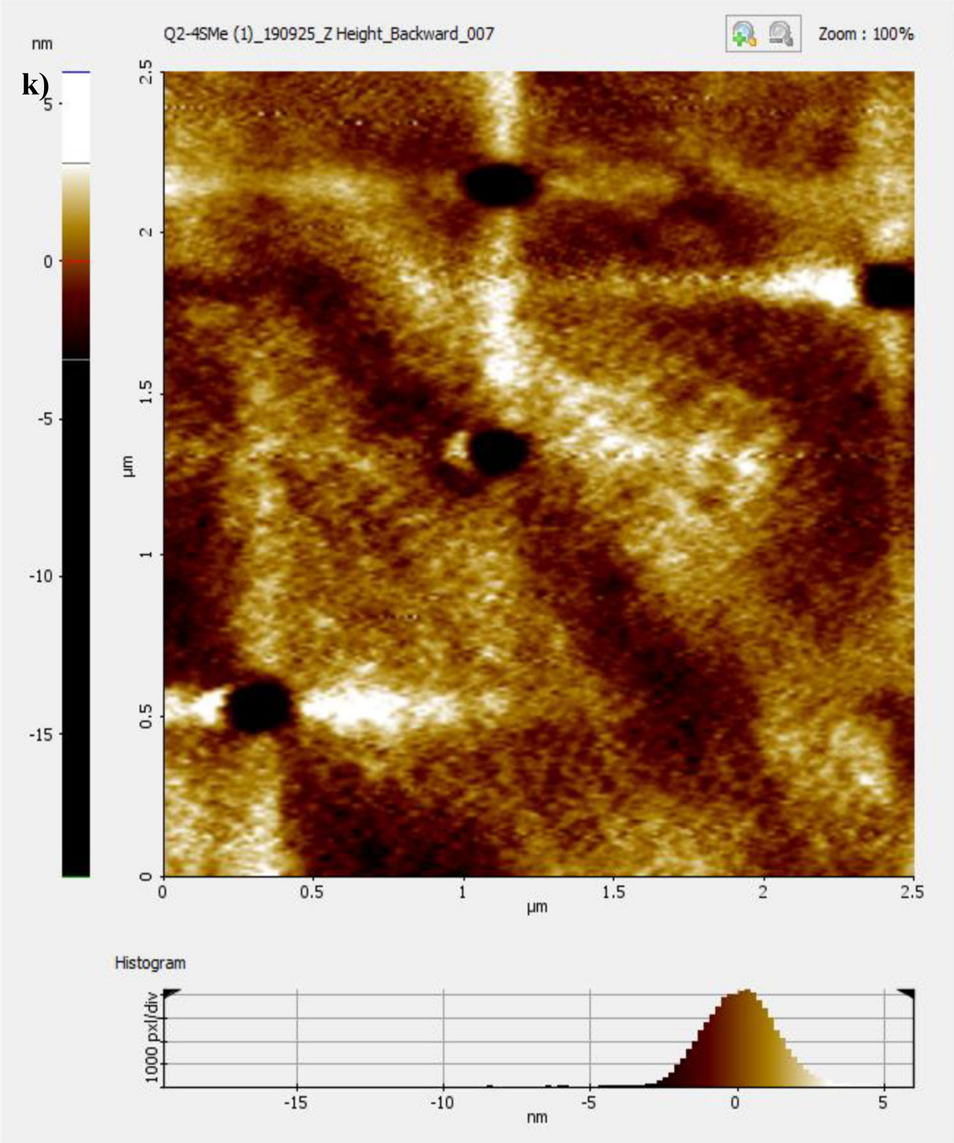
k)
Q2-4SMe (1)_190925_Z Height_Backward_007
Zoom : 100%
nm
µm
Histogram
1000 pxl/div

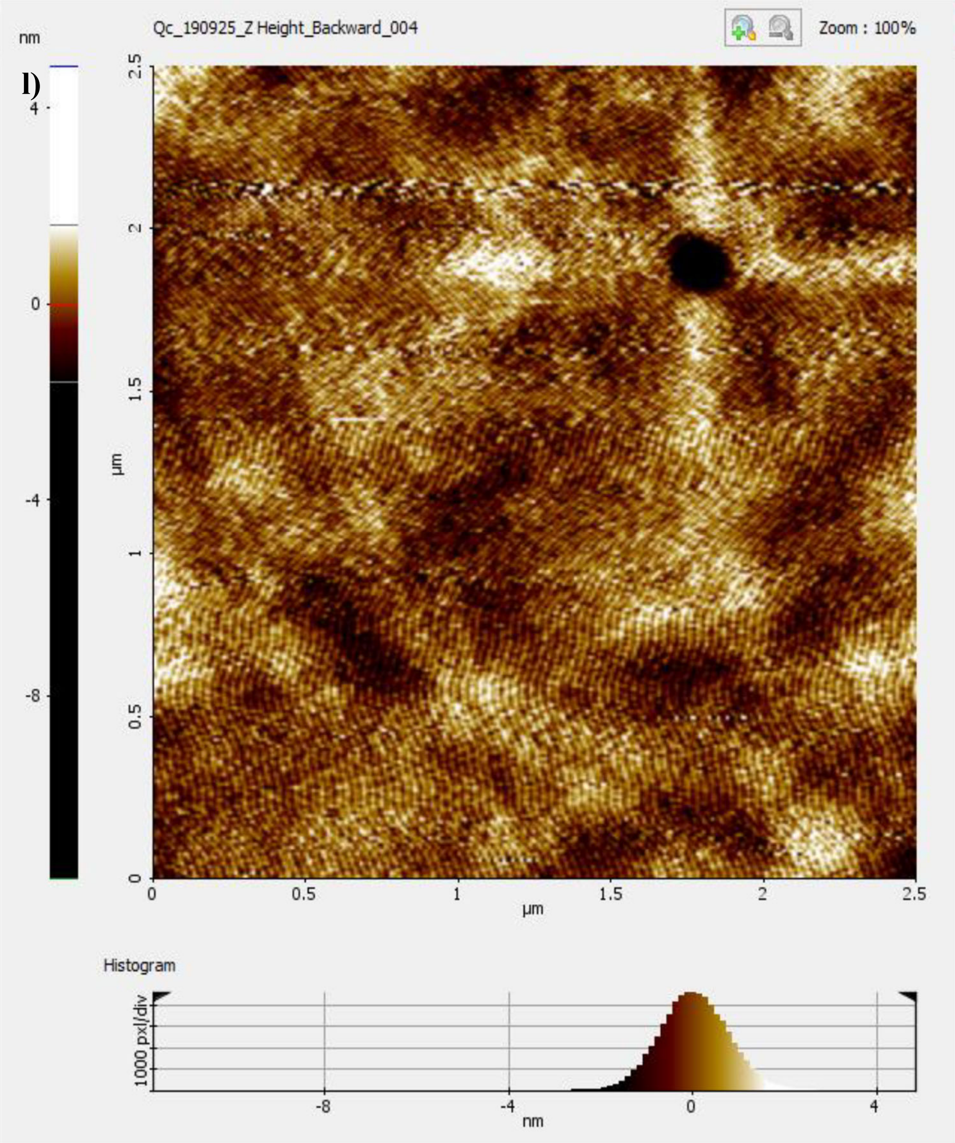
l)
Qc_190925_Z Height_Backward_004
Zoom : 100%
nm
µm
Histogram
1000 pxl/div

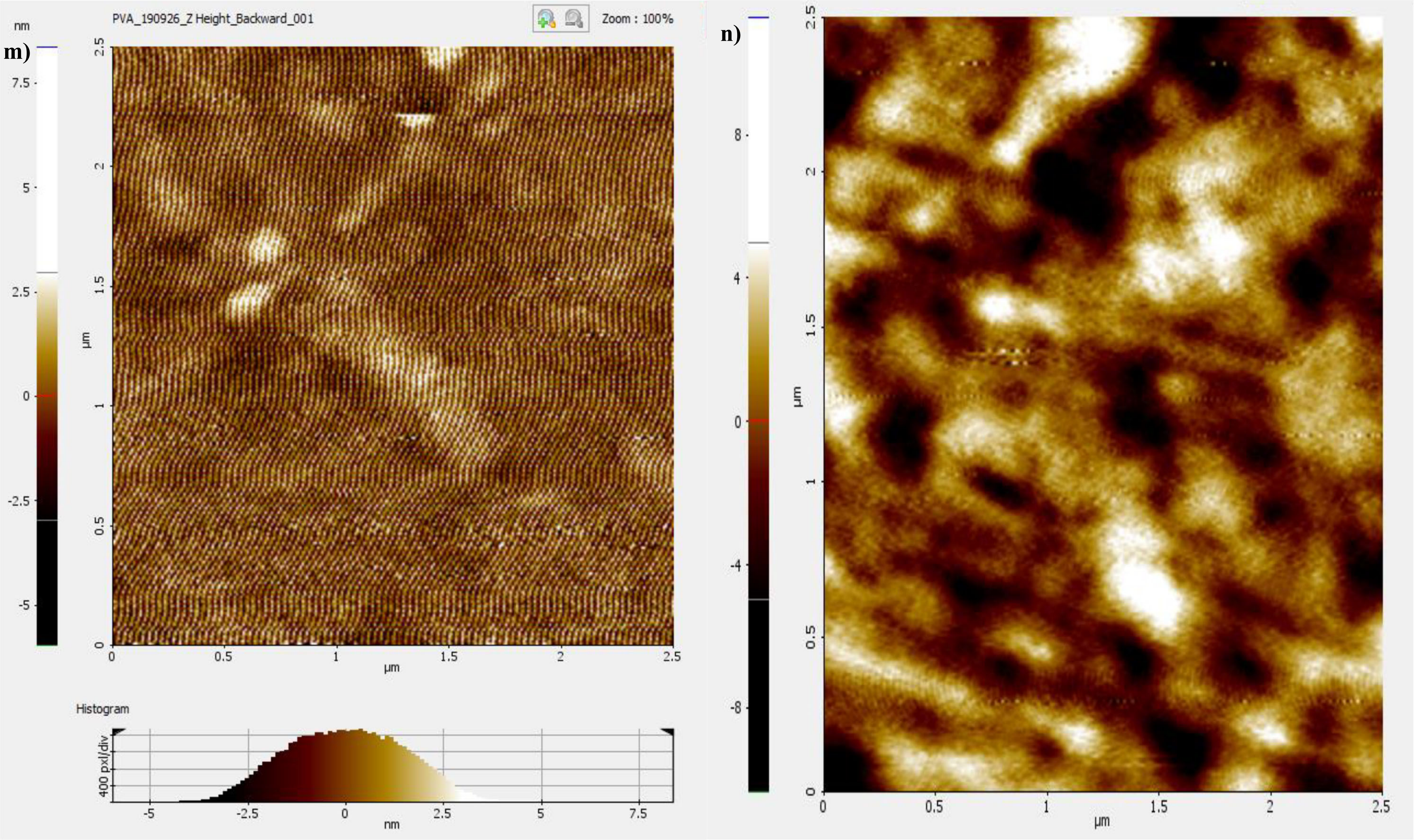

Figure S5. AFM plots of the fabricated films. (a) **1**–PVA, (b) **3a**–PVA, (c) **3b**–PVA, (d) **3c**–PVA, (e) **3d**–PVA, (f) **3e**–PVA, (g) **3f**–PVA, (h) **3g**–PVA, (i) **3h**–PVA, (j) **3i**–PVA. (k) **3j**–PVA, (l) Qc–PVA, (m) PVA, (n) Plastic for packaging.

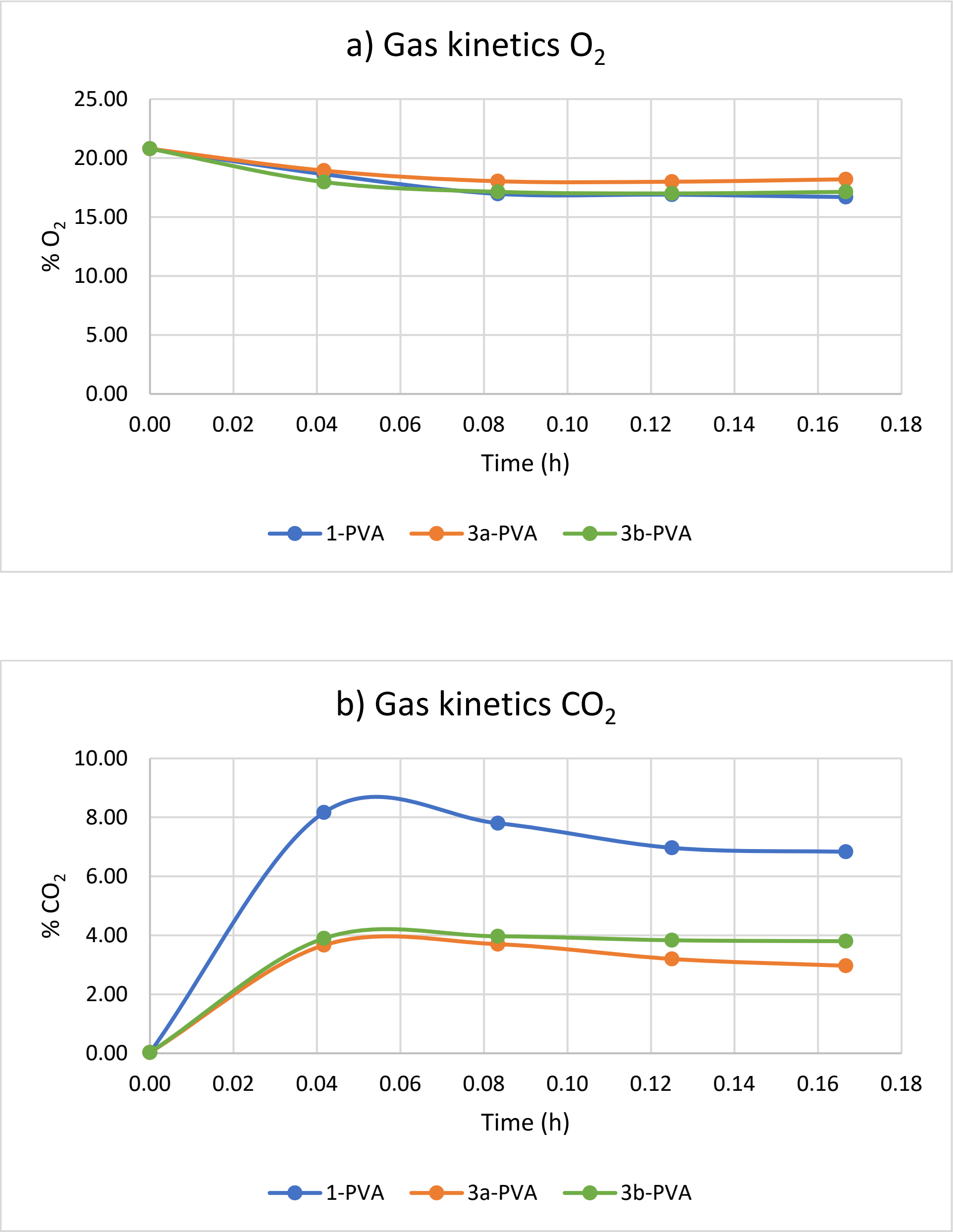

Figure S6. Gas kinetics graphs. (a) O_2, (b) CO_2.

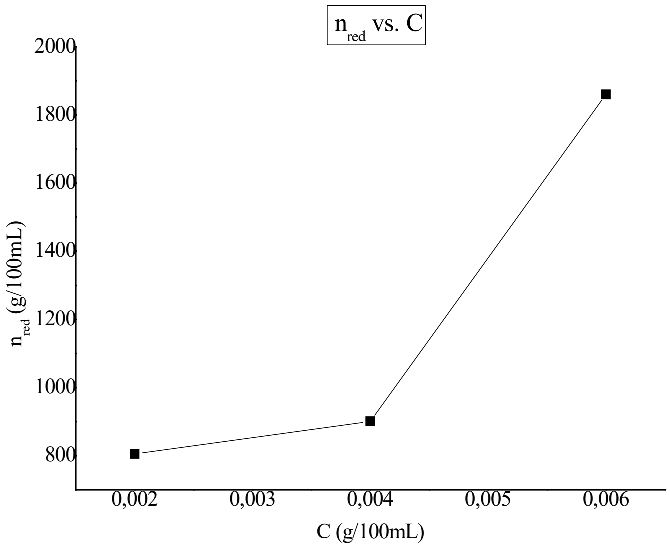

Figure S7. Plot of reduced viscosity vs chitosan concentration.

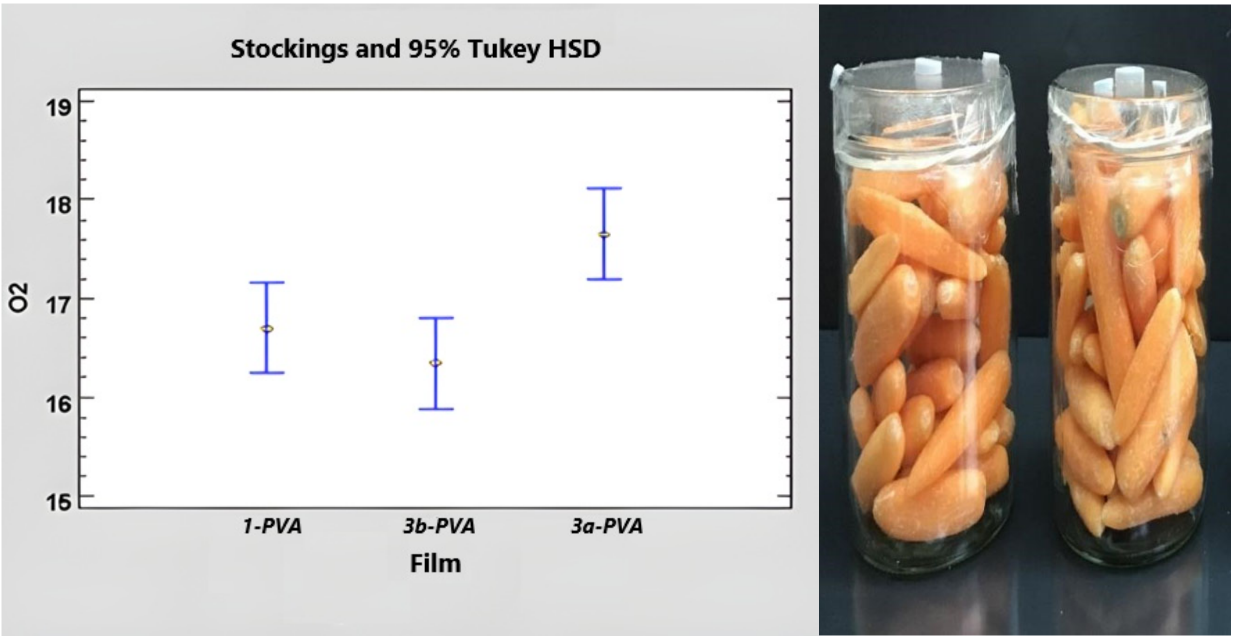

Figure S8. O_2 concentration in jars with carrots packed with films of chitosan **1**-PVA and Schiff bases **3a**–PVA**, 3b**-PVA.

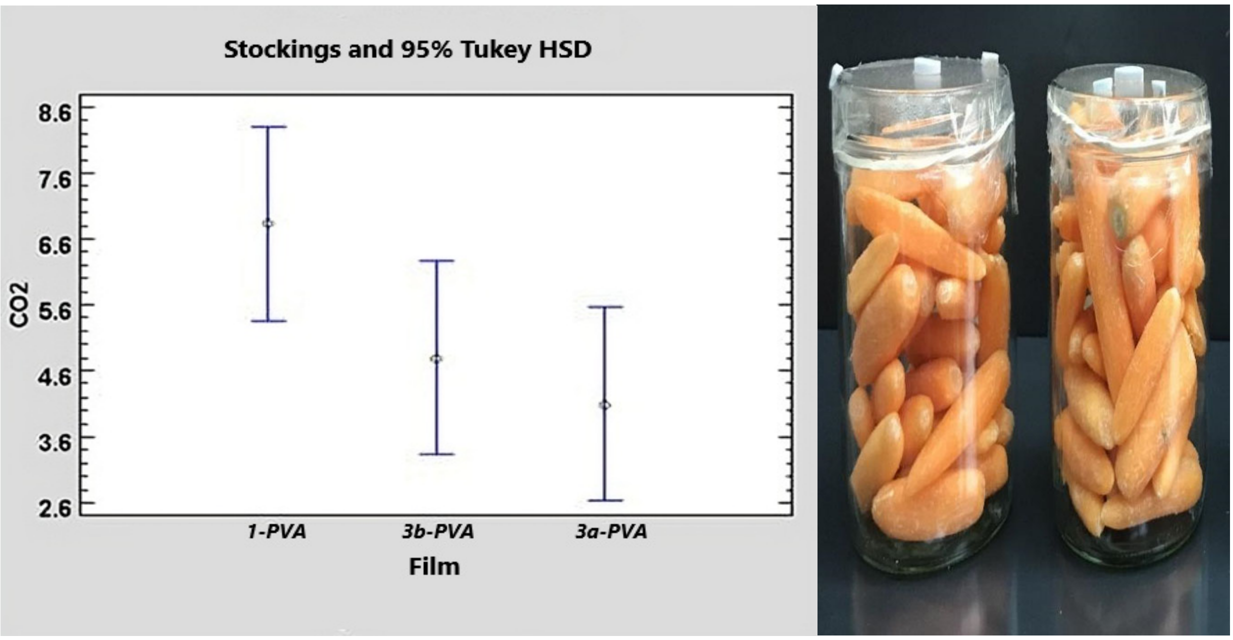

Figure S9. CO_2 concentration in jars with carrots packed with films of chitosan **1**-PVA and Schiff bases **3a**–PVA**, 3b**-PVA.

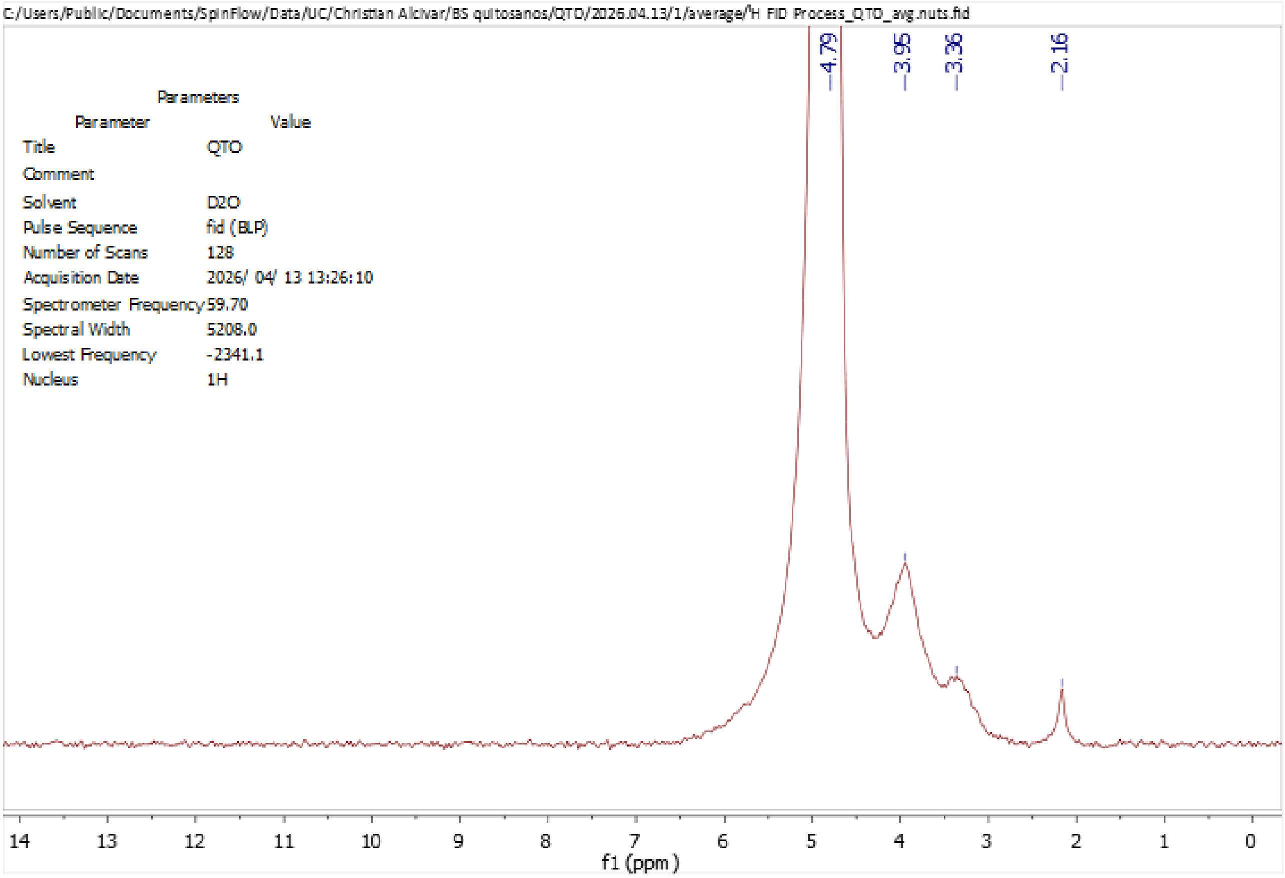

Figure S10. 1H-NMR spectra of **chitosan 1**.

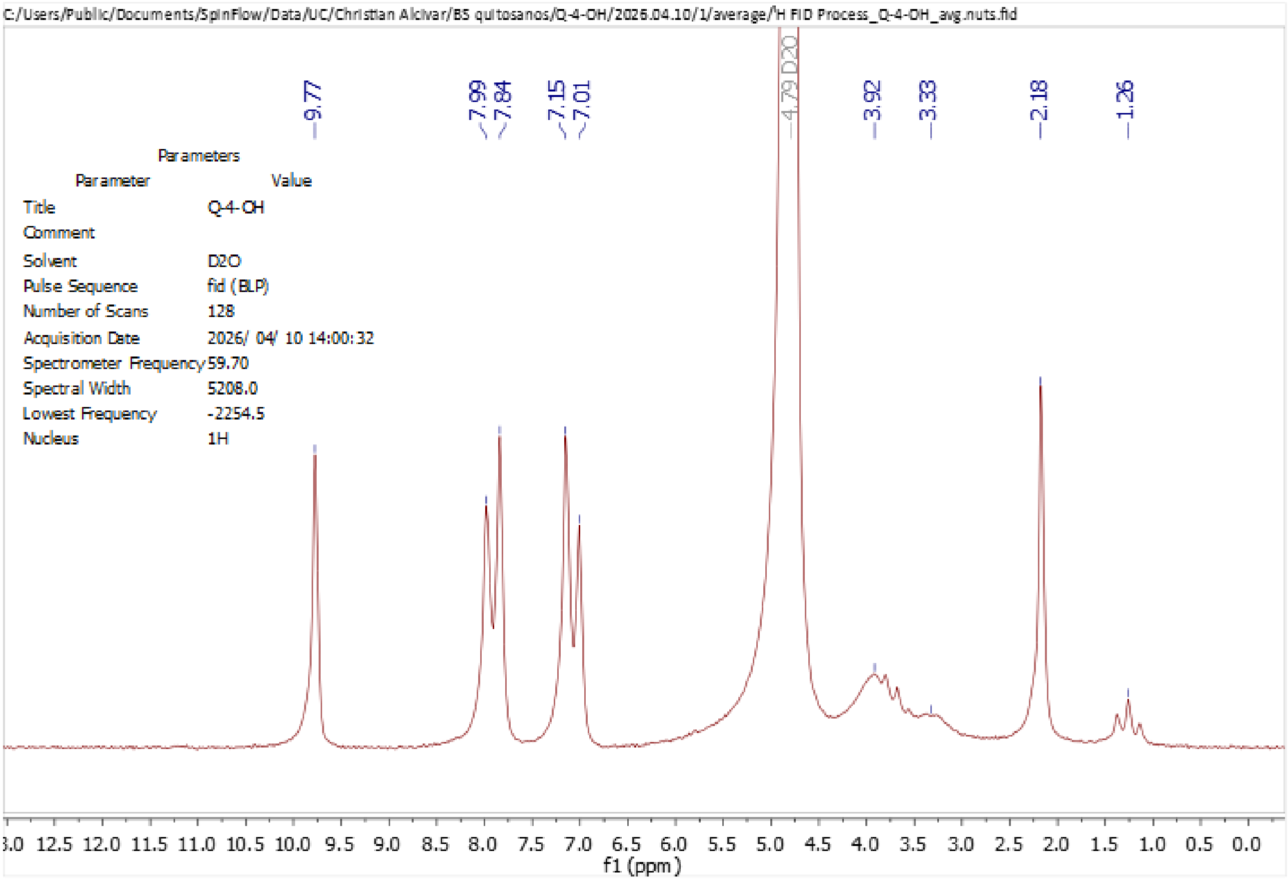

Figure S11. 1H-NMR spectra of **3a**.

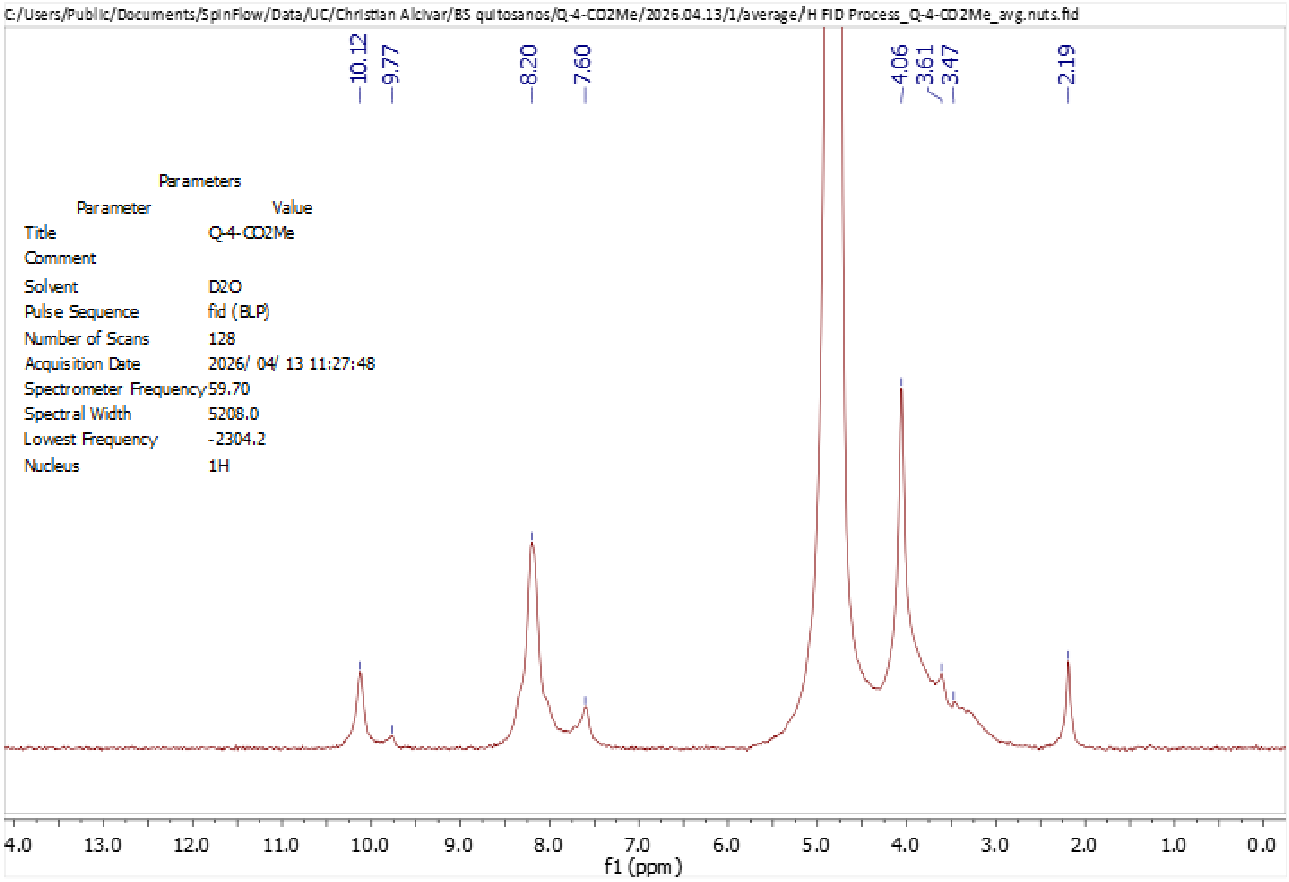

Figure S12. 1H-NMR spectra of **3b**.

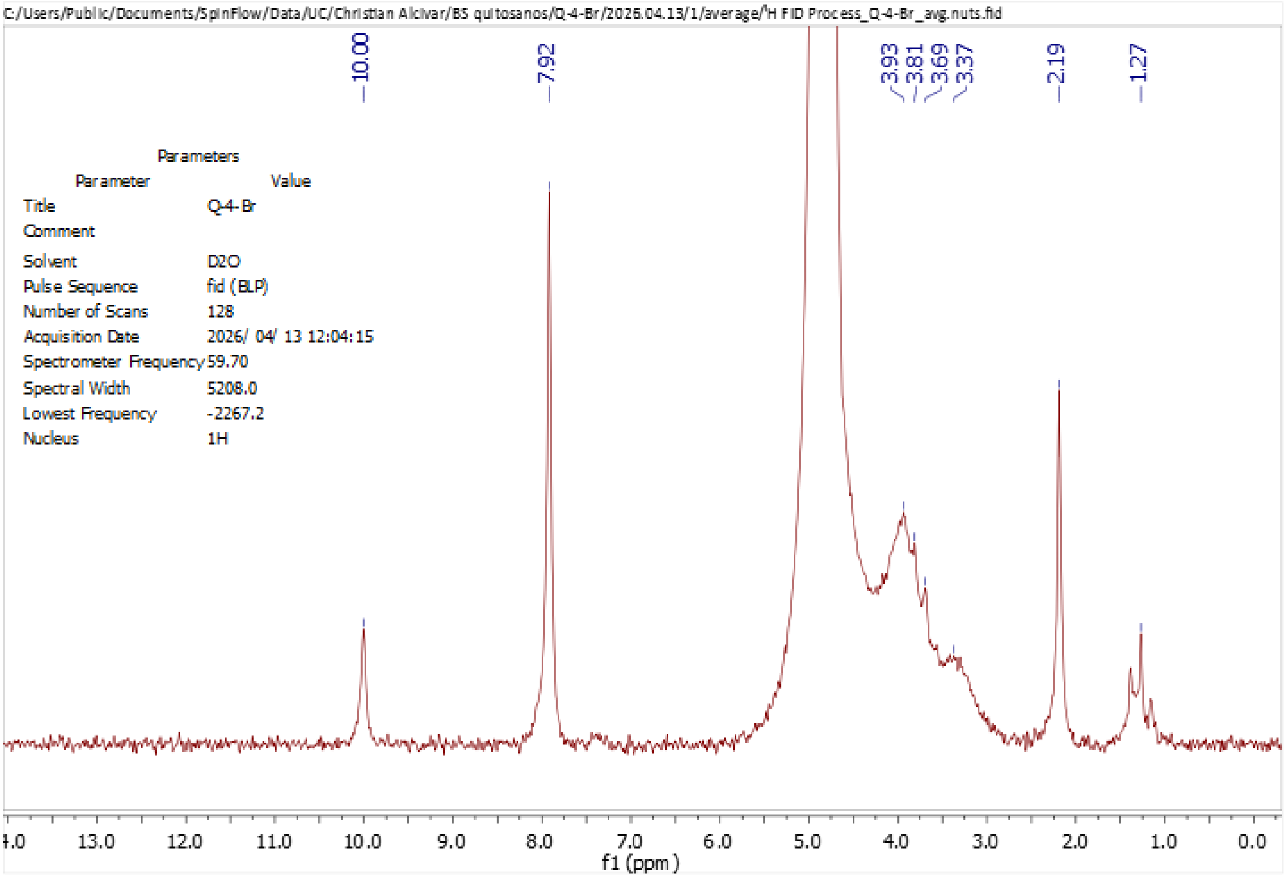

Figure S13. 1H-NMR spectra of **3c**.

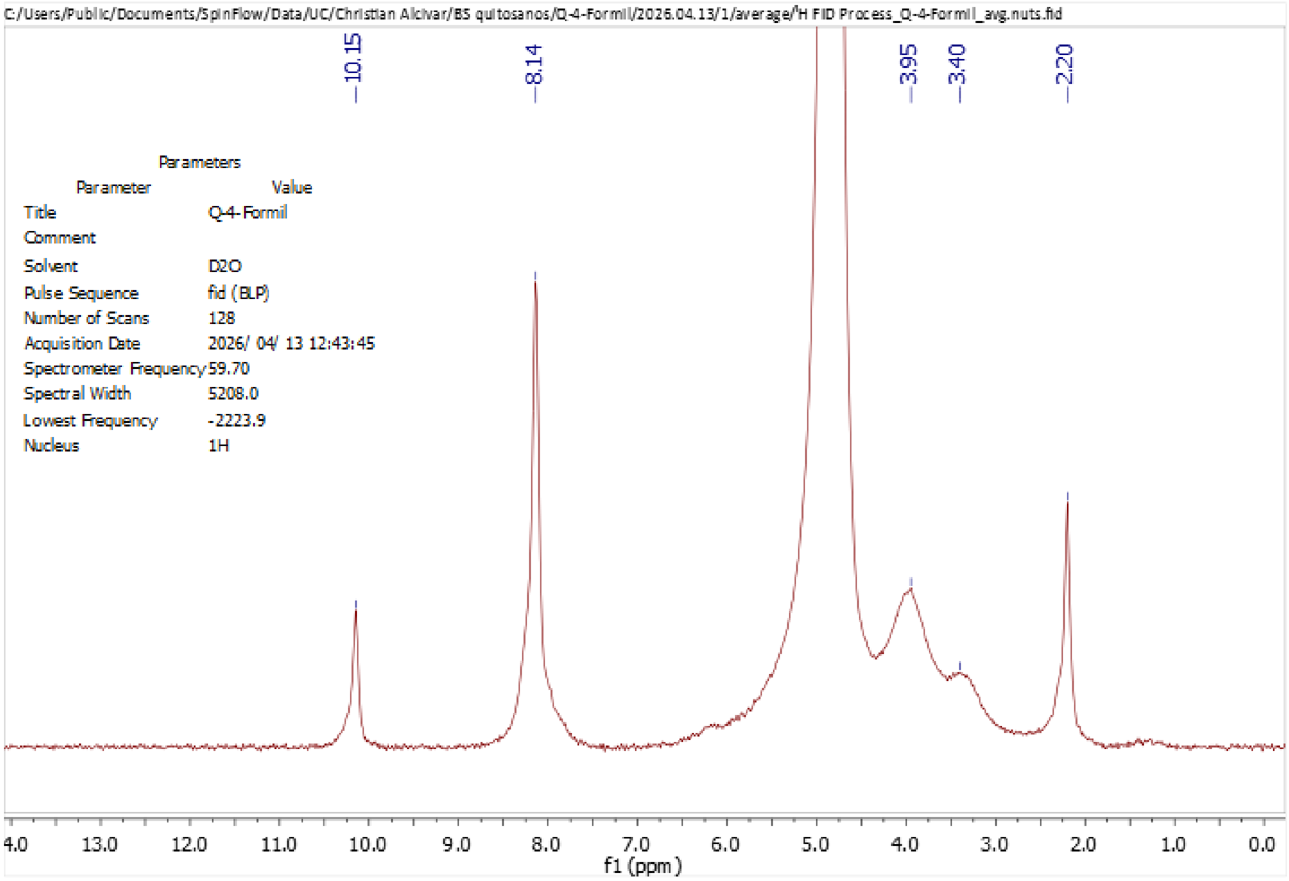

Figure S14. 1H-NMR spectra of **3d**.

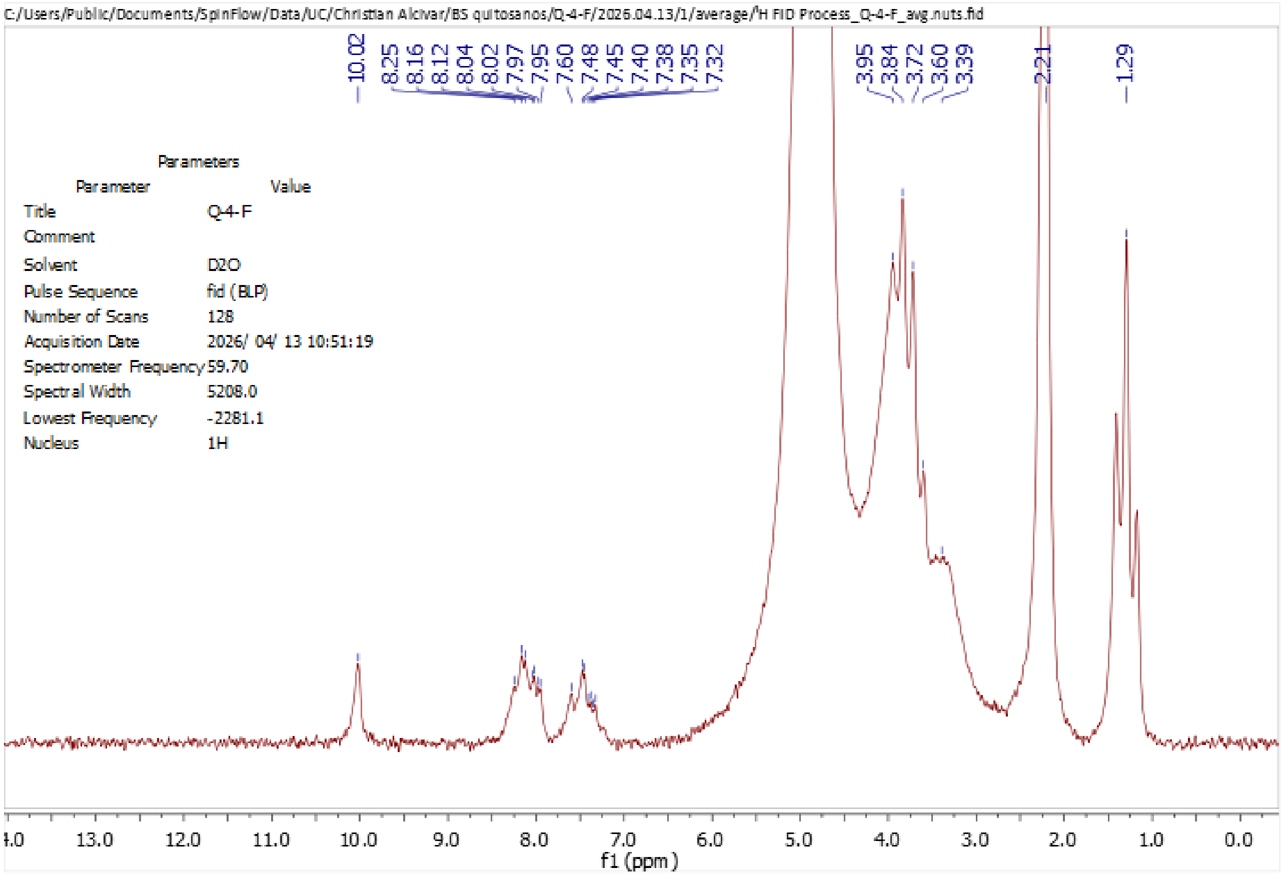

Figure S15. 1H-NMR spectra of **3e**.

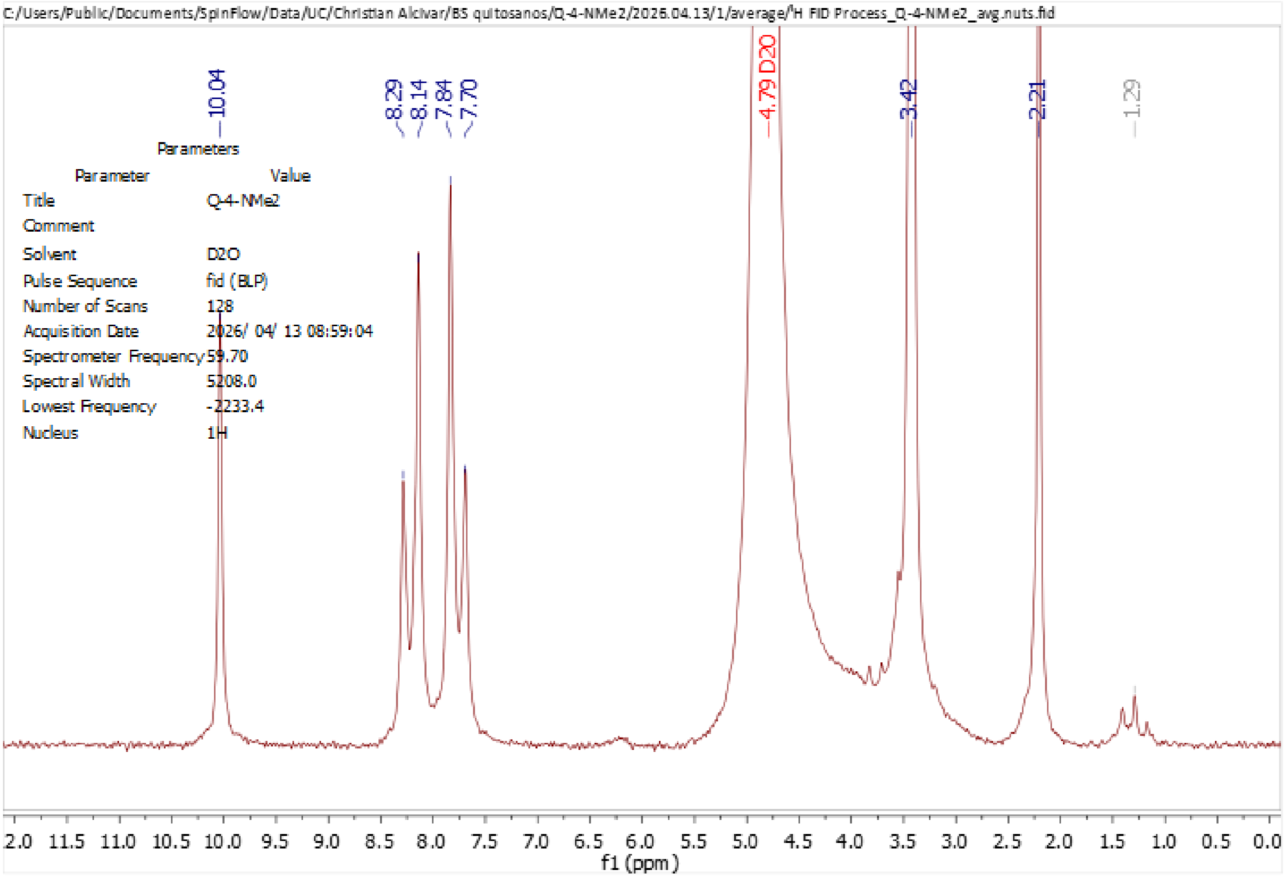

Figure S16. 1H-NMR spectra of **3f**.

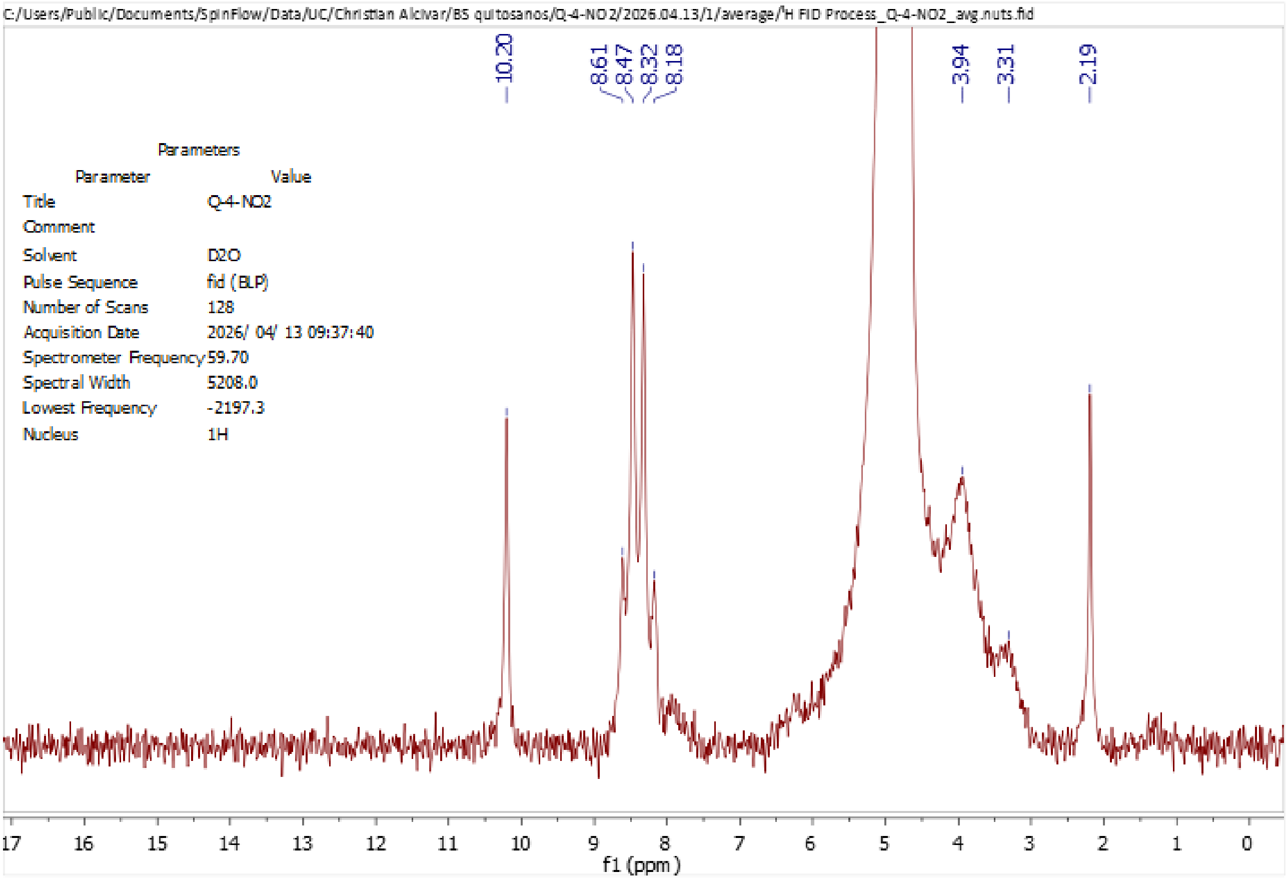

Figure S17. 1H-NMR spectra of **3g**.

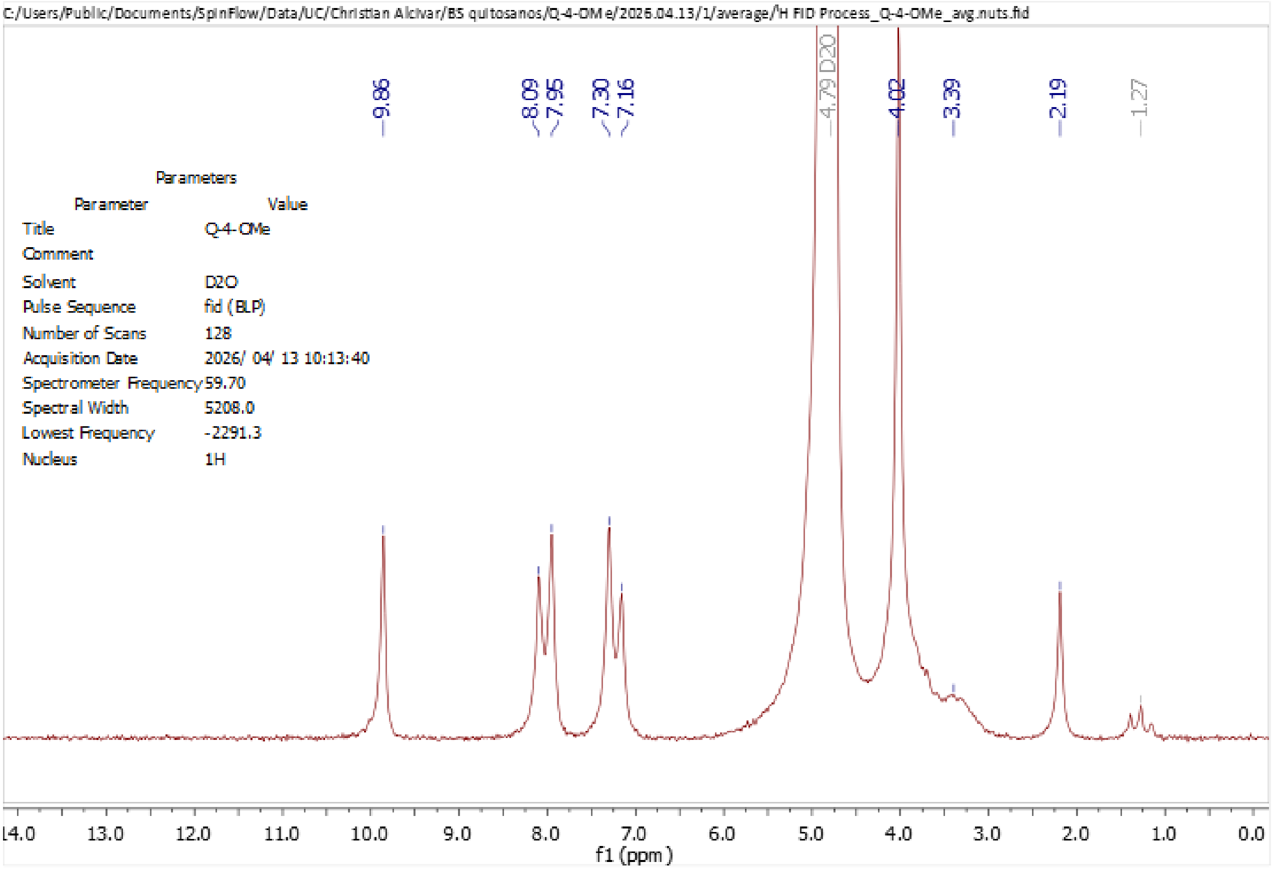

Figure S18. 1H-NMR spectra of **3i**.

Mechanical Properties of Chitosan–Schiff Base/PVA Films

Experimental Details

The mechanical properties of the films were evaluated using a universal testing machine (Shimadzu). Rectangular film specimens were tested at room temperature under a constant crosshead speed of 5 mm/min. The maximum tensile strength (N/mm²), elongation at break (%), and displacement (mm) were recorded. For each sample, at least two independent measurements were performed when possible, and the results are reported as individual values to ensure transparency and reproducibility.

Table S3. Raw mechanical data of chitosan–Schiff base/PVA films

Film	Replicate	Tensile Strength (N/mm^2)	Elongation (%)	Displacement (mm)
1–PVA	1	265.35	64.74	5.18
3c–PVA	1	72.79	233.33	18.67
3c–PVA	2	61.09	436.46	34.92
3a–PVA	1	869.33	421.77	33.74
3a–PVA	2	916.17	369.94	29.60
3g–PVA	1	103.46	538.50	43.08
3g–PVA	2	143.20	571.83	45.75
3e–PVA	1	225.84	441.46	35.32
3d–PVA	1	179.84	455.88	36.47
3d–PVA	2	208.26	453.29	36.26
3b–PVA	1	148.50	362.05	28.96
3b–PVA	2	143.64	428.85	34.31
3f–PVA	1	394.32	378.83	30.31

3f–PVA	2	1713.04	155.88	12.47
3i–PVA	1	198.02	373.81	29.90
3i–PVA	2	125.42	606.78	48.54
Qc–PVA	1	241.62	457.67	36.61
Qc–PVA	2	375.79	571.63	45.73

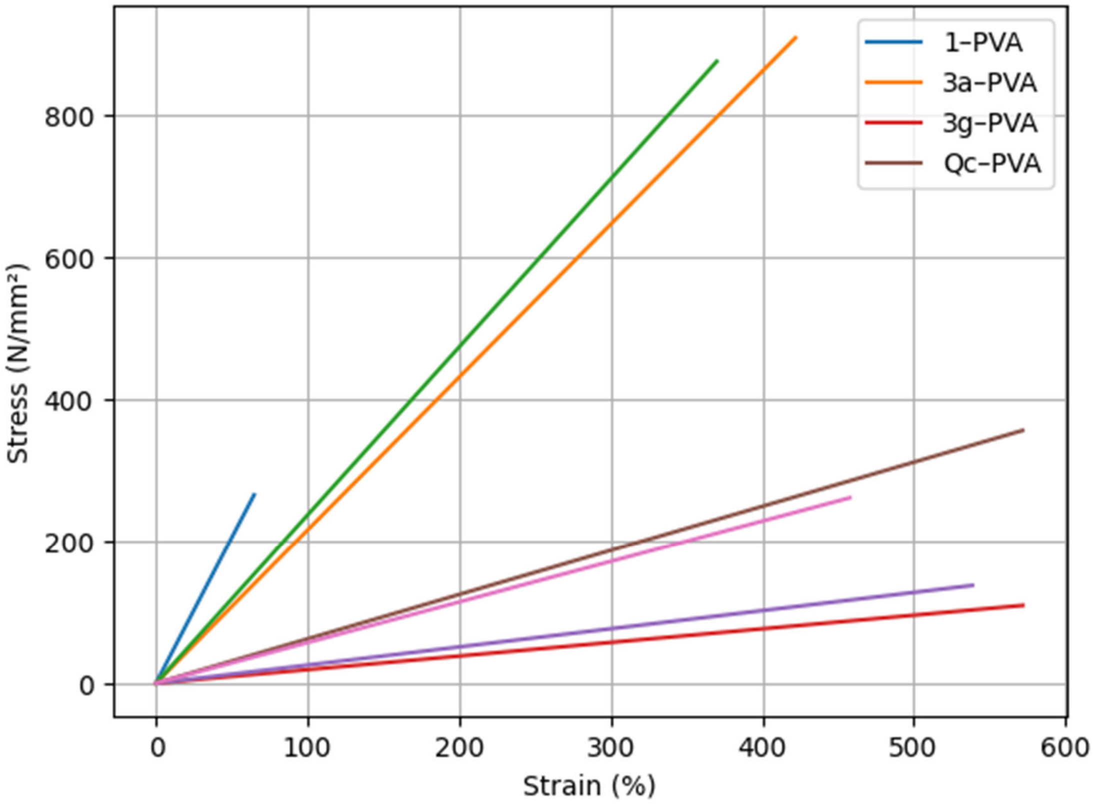

Figure S19. Experimental stress–strain representation of selected films (1–PVA, 3a–PVA, 3g–PVA, and Qc–PVA) based on individual replicate data. Each line corresponds to a single measurement using maximum tensile strength and elongation at break values..
